# Supplementary material for: Dynamics of the compartmentalized Streptomyces chromosome during metabolic differentiation
Source: Nat Commun. 2021 Sep 1;12:5221. doi: 10.1038/s41467-021-25462-1 (PMC8410849; doi:10.1038/s41467-021-25462-1)
Supplement: Supplementary file 1 — Supplementary information [file 41467_2021_25462_MOESM1_ESM.pdf]

Supplementary information

Dynamics of the compartmentalized *Streptomyces* chromosome during metabolic differentiation

Authors

Virginia S. Lioy[1,*], Jean-Noël Lorenzi[1], Soumaya Najah[1], Thibault Poinsignon[1], Hervé Leh[1], Corinne Saulnier[1], Bertrand Aigle[2], Sylvie Lautru[1], Annabelle Thibessard[2], Olivier Lespinet[1], Pierre Leblond[2], Yan Jaszczyszyn[1], Kevin Gorrichon[1], Nelle Varoquaux[3], Ivan Junier[3], Frédéric Boccard[1], Jean-Luc Pernodet[1], Stéphanie Bury-Moné[1,*]

[1]Université Paris-Saclay, CEA, CNRS, Institute for Integrative Biology of the Cell (I2BC), 91198, Gif-sur-Yvette, France.
[2]Université de Lorraine, INRAE, DynAMic, F-54000 Nancy, France
[3]TIMC-IMAG, CNRS - Université Grenoble Alpes, Grenoble, France

[*]Address correspondence to: stephanie.bury-mone@i2bc.paris-saclay.fr; virginia.lioy@i2bc.paris-saclay.fr

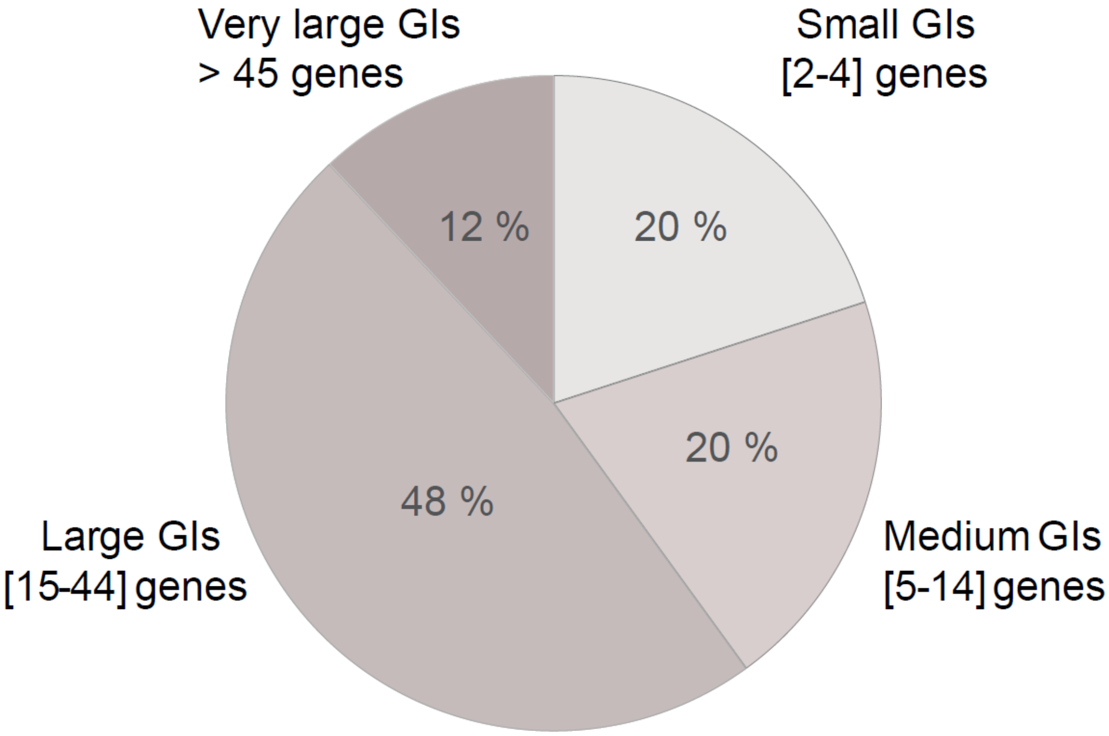

Supplementary Figure 1: Size distribution of *S. ambofaciens* GIs

Using a minimal threshold of 15 CDS within the synteny break in at least one of the compared genome, 50 GIs (composed of 2 to 158 CDSs) and 22 insertion points (≤ 1 CDS within the synteny break) were identified in *S. ambofaciens* ATCC 23877 genome.

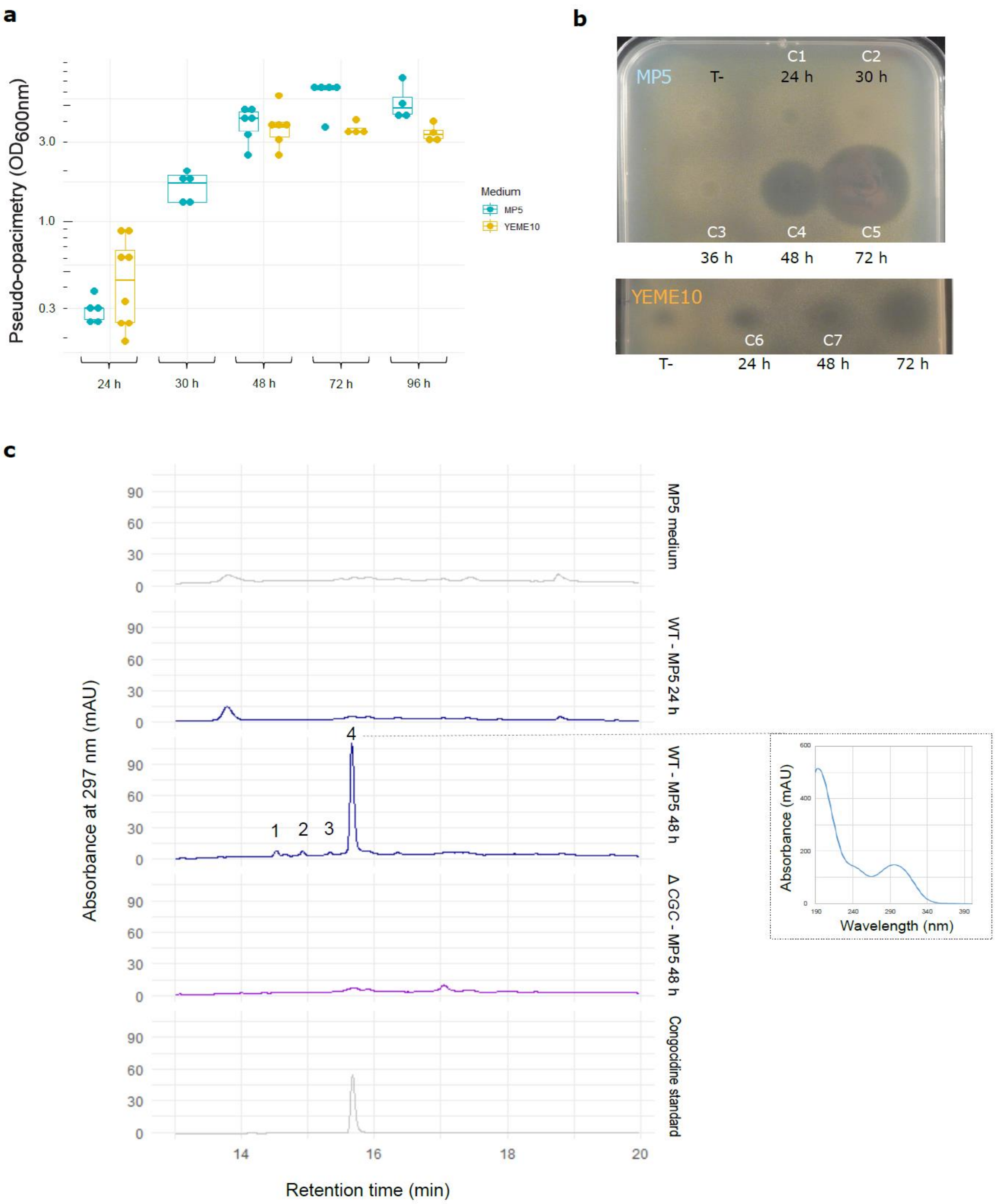

Supplementary Figure 2: The metabolic differentiation of *S. ambofaciens*

a Growth curves of *Streptomyces ambofaciens* in MP5 and YEME10 liquid media. In these media, *S. ambofaciens* grows in a rather dispersed way, so that growth can be monitored by optical density (OD_{600nm}). We refer to “pseudo-opacymetry” because of the presence of some clumps of bacteria. The boxplots represent the first quartile, median and third quartile, each dot corresponding to an independent experiment. The upper whisker extends from the hinge to the largest value no further than 1.5 * the inter-quartile range (IQR, *i.e*. distance between the first and third quartiles) from the hinge. The lower whisker extends from the hinge to the smallest value at most 1.5 * IQR of the hinge.

b Bioassays performed in conditions used in this study. Bioassays were performed against *Micrococcus luteus* with the supernatants of *S. ambofaciens* ATCC 23877

grown in MP5 or YEME10 liquid media and harvested at different time points. Non-inoculated media were used as negative controls (‘T-‘). ‘C1’ to ‘C7’ refer to the name of the studied conditions (Table 1). The width of the plates is 12 cm. The presence of a halo indicates the presence of one or more antibacterial activities. The expression of the SMBGCs encoding known antibiotic is presented in **Supplementary Fig.3c**. Source data are provided as a Source Data file.

c HPLC analyses of the supernatants of *S. ambofaciens* ATCC 23877 during growth in MP5 medium. After 24 h and 48 h of growth, filtered culture supernatants were submitted to HPLC analysis (see Method section for details). Sterile MP5 medium was used as a negative control. The absorbance was monitored at 297 nm. After 48 h of growth, the presence in the culture supernatants of several peaks (highlighted by numbers) that are absent after 24 h of growth illustrates the process of metabolic differentiation of *S. ambofaciens.* The peak # 4 around 15.6 min presents the same retention time and absorbance spectrum absorbance (one representative result in the islet) than an authentic congocidine standard and is absent in the culture supernatant of a strain with the congocidine cluster deleted (‘Δ*CGC*’, lab collection). This peak therefore corresponds to congocidine. The results correspond to the mean of five independent experiments per strain. Source data are provided as a Source Data file.

Supplementary Figure 3

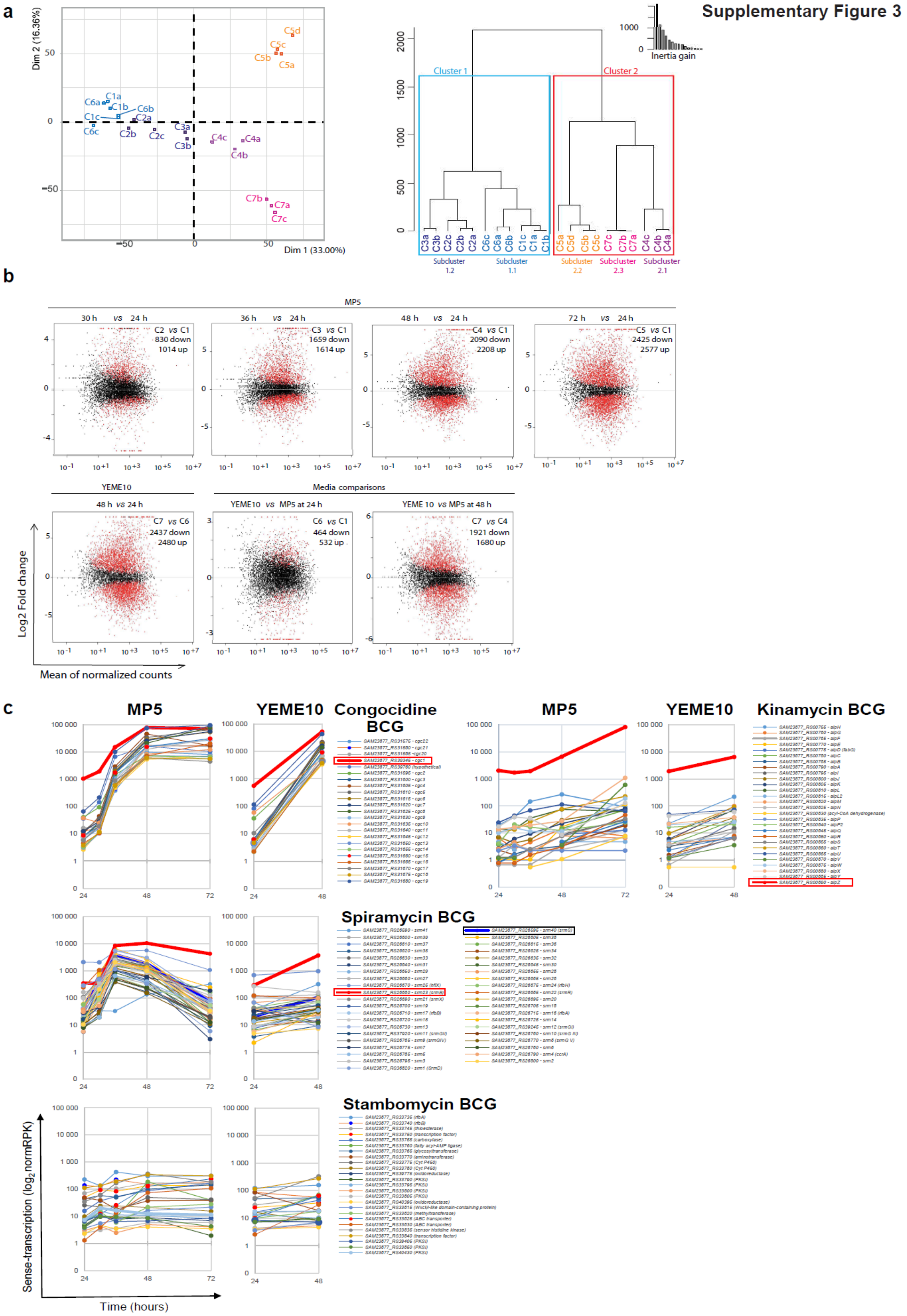

Supplementary Figure 3: The metabolic differentiation of *S. ambofaciens* analyzed at the transcriptional level

a Multidimensional analyses of the studied conditions. Left panel - Axes 1 and 2 of the principal component analysis (PCA) of the whole data set (21 samples), with percentages of variance associated with each axis. The numbers indicate the condition (see Table 1) with a distinct letter (a, b, c, d) for each replicate. Right panel - Ascending hierarchical classification (Euclidean distance, Ward criterion) and its inertia gain. The conditions form two clusters (1 and 2) and five subclusters which are boxed. The cluster 1 corresponds to the earliest growth time points (24 h, 30 h and 36 h) in which no antibiotic production was detected by bioassay (**Supplementary Fig.2b**). The cluster 2 corresponds to conditions associated to metabolic differentiation and the latest growth time points. Its sub-clusters separate owing the growth time and medium: 48 h in MP5 (subcluster 2.1), 72 h in MP5 (subcluster 2.2), 48 h in YEME10 (subcluster 2.3).

b MA-plot comparing transcriptomes over growth and over media. The MA-plot represents the log ratio of differential expression as a function of the mean intensity for each pairwise comparison. The differentially expressed features are highlighted in red, and the number of up- and down-regulated genes indicated on each graph. Triangles correspond to features having a too low/high $\log_2$ (fold change) to be displayed on the plot.

c Transcriptomes over growth of the four biosynthetic gene clusters encoding all known antibacterial activities of *S. ambofaciens*. Genes expressed at the highest level are boxed in red for the congocidine, spiramycin and kinamycin BGCs. They correspond to *cgc1* (SAM23877_RS39345, encoding a transcriptional regulator), *srmB* (SAM23877_RS26680, encoding the ABC-F type ribosomal protection protein SrmB) and *alpZ* (SAM23877_RS00890, encoding a transcriptional regulator) genes, respectively. The gene encoding SrmS (SAM23877_RS26595), the transcriptional regulator of the spiramycin BGC, is boxed in black. The dots correspond to the mean number of DESeq2 normalized reads per kb (normRPK) of the replicates, and are joined by a line for clarity (but they do neither report a real observation nor a model).

Supplementary Figure 4

a

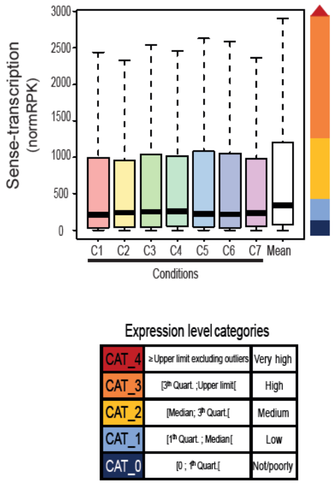

b

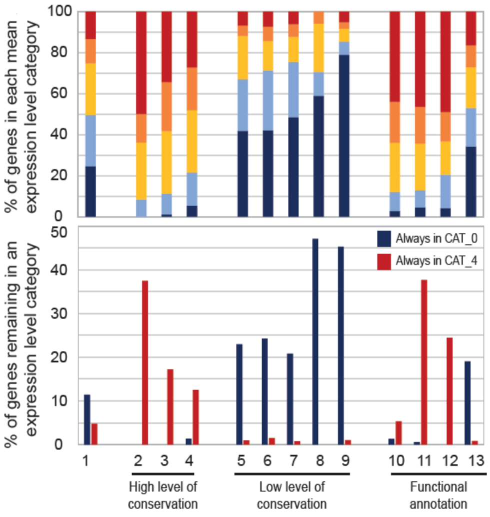

	Proportion of genes always expressed at very high level (CAT_4) compared to the whole set of chromosomal genes		Proportion of genes always not/poorly expressed(CAT_0) compared to the whole set of chromosomal genes	
	Odds ratio	*p* value	Odds ratio	*p* value
1. Chr.				
2. Actino.Sign.CDSs	11.9	< 2.2e-16	0.0	2.6e-04
3. Core CDSs	4.2	< 2.2e-16	0.0	< 2.2e-16
4 Persist $_{>0.95}$CDSs	2.9	< 2.2e-16	0.1	< 2.2e-16
5. GIs	0.2	5.5e-12	2.3	< 2.2e-16
6. Strain -specific CDSs	0.3	2.6e-02	2.5	6.1e-07
7. pSAM1	0.2	3.3e-02	2.0	2.2e-03
8. pSAM2	0.0	1	6.9	2.8e-04
9. Prophage	0.2	1.4e-01	6.4	3.0e-16
10. Func. RNAs (-rRNAs)	1.1	7.8e-01	0.1	2.7e-03
11. Transl. CDSs	12.0	< 2.2e-16	0.04	3.2e-08
12. NAPSF genes	6.5	2.9e-06	0.0	5.2e-03
13. SMBGCs	0.2	7.4e-05	1.8	4.7e-05

c

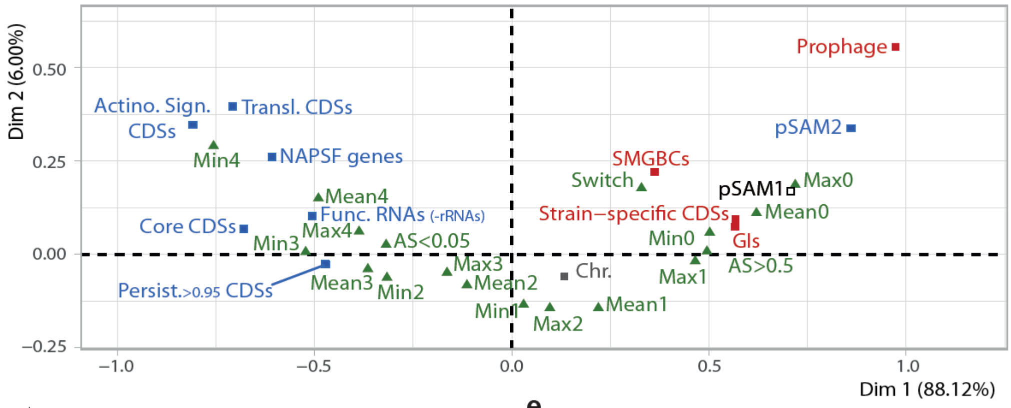

d

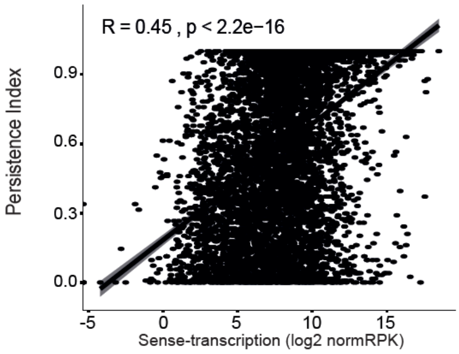

e

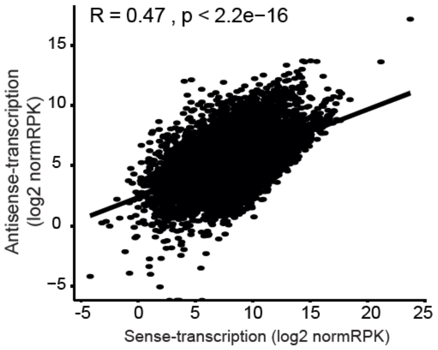

f

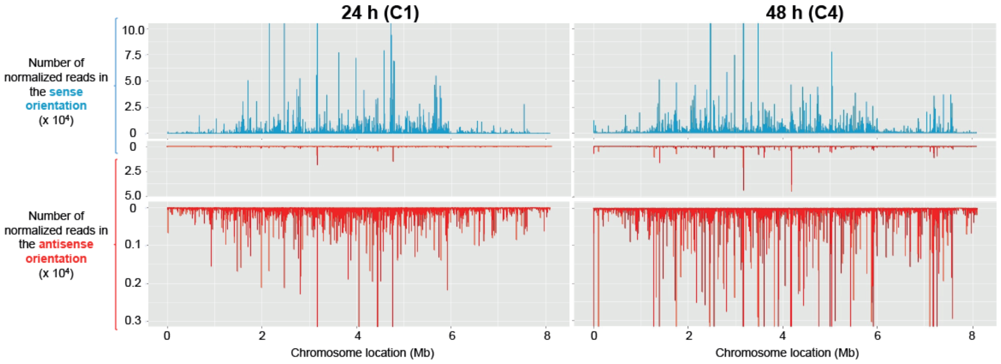

Supplementary Figure 4: The dynamic of *S. ambofaciens* transcriptome

a Categories of gene expression level. The boxplot represents the distributions of the read counts per gene (normalized by DESeq and on gene size, RPK) individually for each tested condition and as the mean expression of each gene in all tested conditions. The genes were classified owing their expression level by considering the distribution parameters as indicated. The boxplots represent the first quartile, median and third quartile. The upper whisker extends from the hinge to the largest value no further than 1.5 * the inter-quartile range (IQR, *i.e*. distance between the first and third quartiles) from the hinge. The lower whisker extends from the hinge to the smallest value at most 1.5 * IQR of the hinge. Outliers are not represented.

b Pattern of gene expression depending on genome features of interest. The genes were classified owing their mean (upper panel), their minimal or their maximal (lower panel) expression level in all studied conditions. The table presents the statistical analysis of the data (two-sided Fisher's exact tests for count data).

c Multidimensional analysis (overlay factor map) of genomic and transcriptomic data. The squares correspond to the raw labels (genome features, see **Fig.1** for abbreviations). The triangles correspond to the column labels (*e.g*. category of level of mean, maximal, minimal sense-transcription, antisense index < 0.05, antisense index > 0.5). The closer the squares or triangles are to the origin of the graph, the closer they are likely to be to the mean data. Proximity between triangles or between squares indicates similarity. A small angle (*e.g*. less than 30°) connecting a triangle and a square to the origin indicate that they are probably associated. If they are located at the opposite, they are probably negatively associated. The proximity between the variables 'SMBGCs' and 'Switch' is due to the fact that a high proportion (19.3 %) of the SMBGC genes switched on at the transcriptional level in at least one studied condition (compared to 4.4 % at the level of the whole genome, odds ratios 5.2, *p*-value < $2.2.10^{-16}$; 7.6 % of genes within GIs, odds ratios 2.9, *p*-value $2.2.10^{-9}$, two-sided Fisher's exact tests for count data).

Other abbreviations: 'AS < 0.05' and 'AS > 0.5': very low (below 0.05) or very high (above 0.5) antisense-transcription indices; 'Max', 'Mean' and 'Min': maximal, mean and minimal expression levels in all studied condition, from the lowest category ('0') to the highest ('4'); 'Switch': switch from CAT_0 to CAT_3 or more.

d & e Correlation between gene persistence and transcription in sense (**d**) and antisense (**e**) orientation. This correlation was analyzed by a Spearman's rank correlation test. Gene transcription corresponds to the number of DESeq2 normalized reads per kb (normRPK).

f Mapping of sense- and antisense- reads along the chromosome during growth in MP5 medium. The normalized counts [transcripts per million, normalized considering the total (sense- plus antisense-) transcription] were mapped on the *S. ambofaciens* chromosome. Please note that transcription in the antisense orientation is presented on two different scales.

Supplementary Figure 5

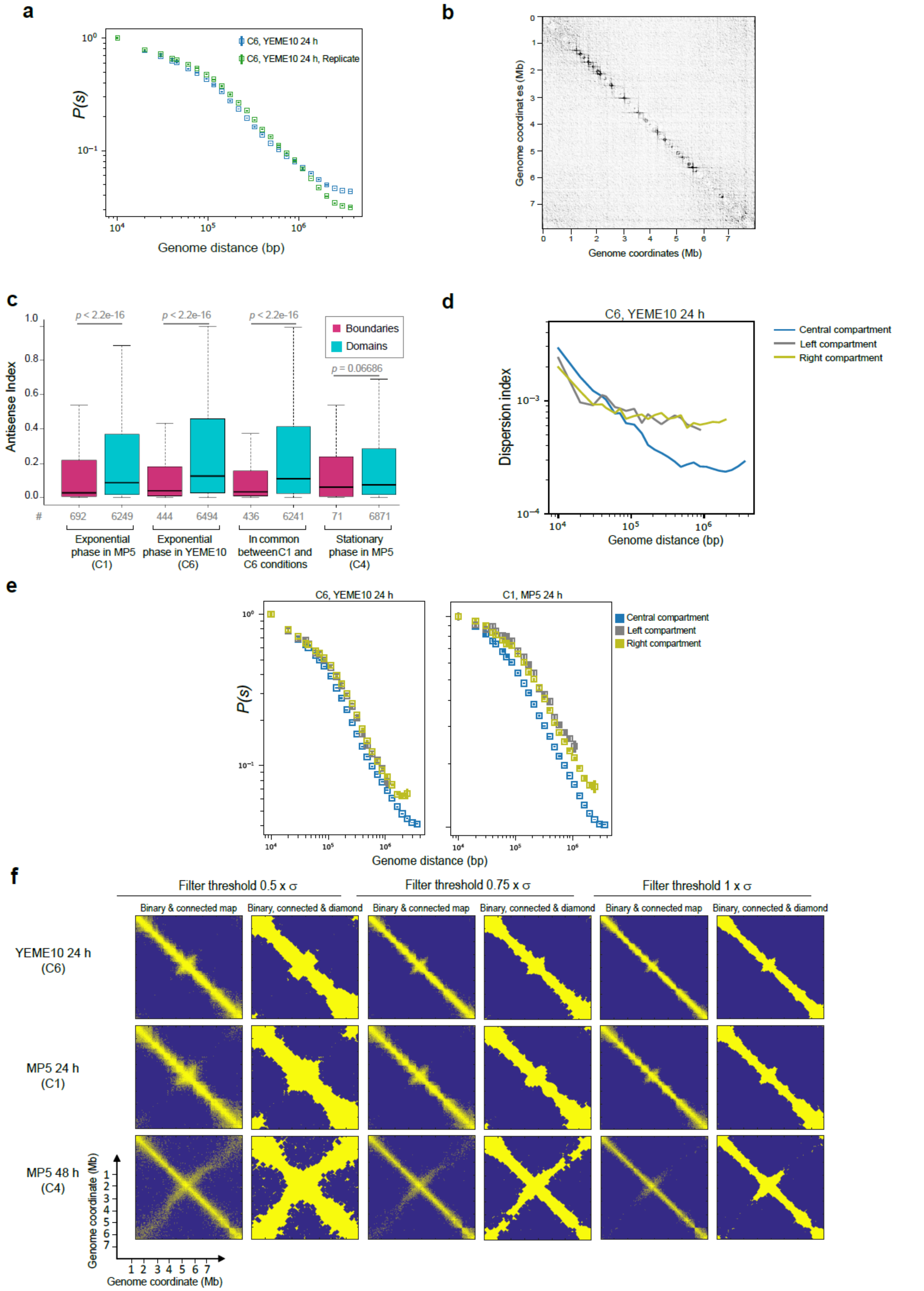

Supplementary Figure 5: Analyses of chromosome structure and its correlation with transcription

a Contact probability plotted as a function of genomic distance in YEME10 at 24 h. The plot represent the intra-chromosomal contact probability, *P(s)*, for pairs of loci separated by a genomic distance on the chromosome. Results of two replicates are presented.

b Derivative of the contact matrix for WT cells in YEME10 at 24 h. The map shows for each genome locus the tendency of contact frequencies to go downwards or upwards. Boundaries are visualized as the black vertices of the squares along the diagonal. Regions with no upstream/downstream bias show no squares (*e.g*. terminal regions).

c Boxplot presenting the antisense index over growth depending of genes and their structural location. The distribution of the antisense index is presented depending on the location of the genes. The antisense indexes in C1 condition of the genes at the boundaries or domains in common between C1 and C6 conditions ('Common C1 & C6') are also presented. The boxplots represent the first quartile, median and third quartile. The upper whisker extends from the hinge to the largest value no further than 1.5 * the inter-quartile range (IQR, *i.e*. distance between the first and third quartiles) from the hinge. The lower whisker extends from the hinge to the smallest value at most 1.5 * IQR of the hinge. Outliers are not represented. The *p*-values of two-sided Wilcoxon rank sum tests with continuity correction (boundaries *versus* domains) are indicated. The number of analyzed genes ('#') per condition is indicated. Please note that the antisense index was not calculated when no reads were detected, either in sense or antisense orientation, in all replicates.

d Dispersion index (DI) of compartments plotted as a function of genomic distance in YEME10 after 24 h of growth. The DI reflects the range of variations relative to the mean value as a function of the genome distance for the contact maps. Long-range DNA contacts within the terminal compartments (> 100 kbp) are more variable than within the central compartment.

e Contact probability of the compartments plotted as a function of genomic distance.

f Contact map analysis of the primary and secondary diagonals. Contact maps were obtained after setting the threshold for significant interactions at 0.5 x and 1.0 x the standard deviation above the median to the original 3C-contact maps obtained in exponential (24 h in MP5 and YEME10 media) and stationary (48 h in MP5 medium) phase. Contact frequencies above or below the threshold were assigned a value of 1 or 0, respectively, generating a binary contact map in which significant interactions are represented in yellow, whereas non-significant interactions are represented in blue. Connected binary maps were generated by connecting 10 elements considered as significant using a diamond shape of 15 to fill out the empty points comprised by the connected elements[1,2].

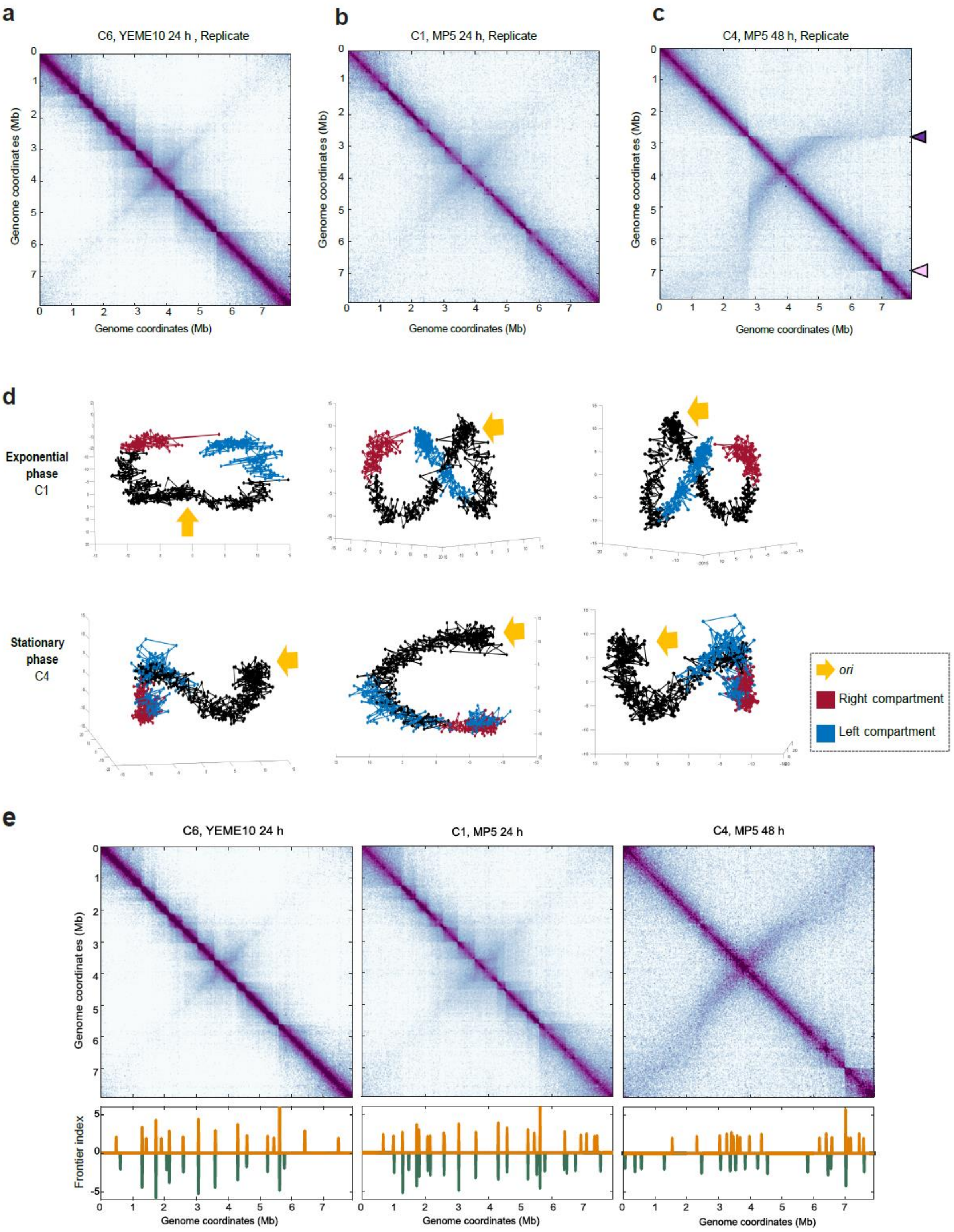

Supplementary Figure 6: Contact maps and 3D-models for *S. ambofaciens*

a Replicate of cells grown 24 h in YEME10 growth medium, C6 condition

b Replicate of cells grown 24 h in MP5 growth medium, C1 condition

c Replicate of cells grown 48 h in MP5 growth medium, C4 condition. The violet and pink arrows indicate the location of boundaries formed at desferrioxamin and congocidine BGCs, respectively.

d 3D-models of *S. ambofaciens* genome generated by ShRec3D software[3]

e Results of the frontier index analysis including peaks that do not form boundaries. This panel presents the frontier index analysis of the 3C-maps presented in **Fig.3** and **Fig.4**, showing single peaks that where not considered as indicative of a boundary. At each locus, two indices are computed, reflecting the intensity of the loss of contact frequencies when going downwards (green peaks) or upwards (orange peaks), respectively, to the locus. A boundary is defined as any bin in which there is a change in the right bias of contacts towards the left bias (± 2 bin, green and orange peaks, respectively). The detection of bins with only an upstream or a downstream significant peak reflects the intrinsically noisy nature of 3C-seq data.

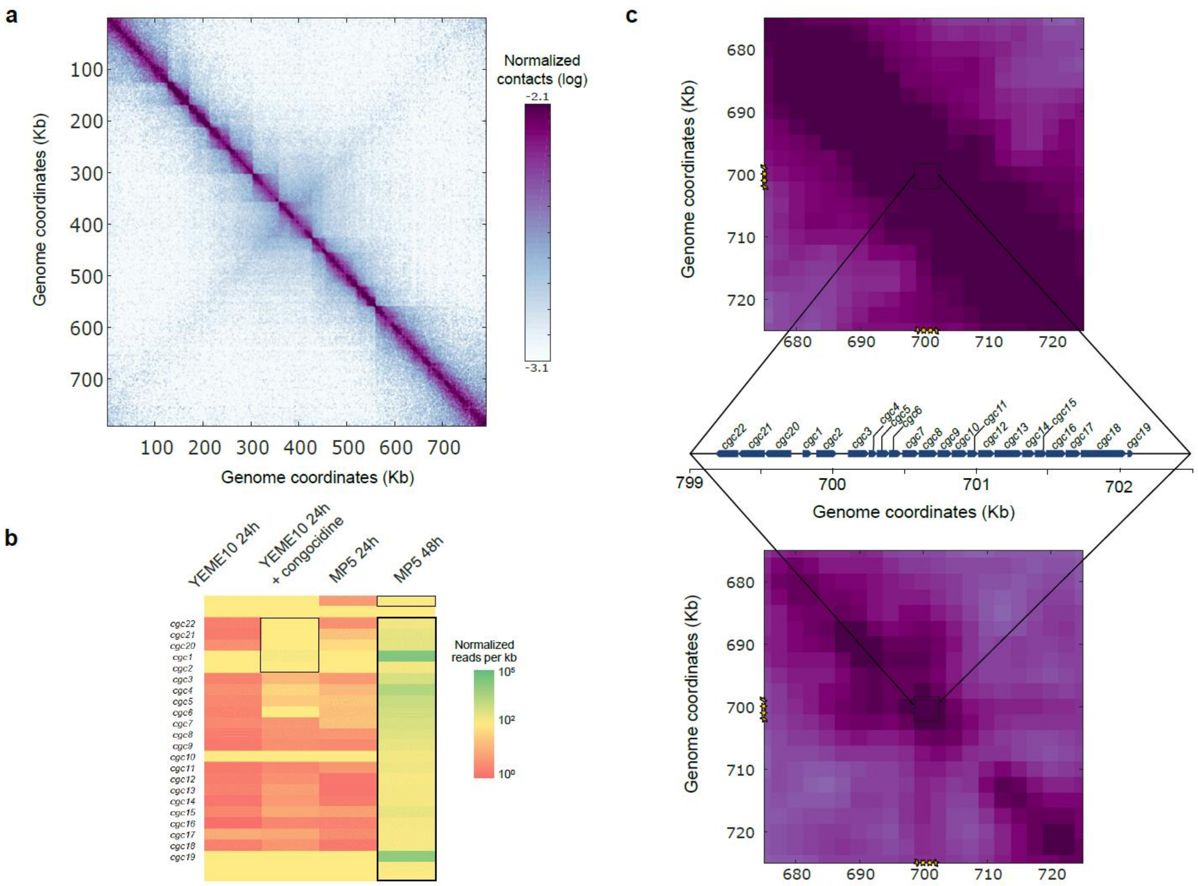

Supplementary Figure 7: Impact of exogenous congocidine on congocidine biosynthetic gene cluster architecture and expression

a Contact map after 24 h in YEME10 growth medium in the presence of exogenous congocidine. 3C-seq was performed on *S. ambofaciens* grown in exponential phase in YEME10 medium supplemented with 1 µg/ml congocidine. The color scale reflect the frequency of the contacts between genome loci, from white (rare contacts) to dark purple (frequent contacts).

b Heatmaps of the congocidine BGC transcription in different growth conditions. Genes whose expression is statistically regulated (*p*-value < 0.05, compared to the control condition, *i.e.* 24 h in the same growth medium) are boxed.

c Focus on the genomic region (50 kb) encompassing the congocidine cluster contact maps (squares) in YEME10 growth conditions in the absence (top) or presence (bottom) of exogenous congocidine. The focus was performed on the 3C-contact maps presented in **Fig.3a** (YEME10) and in panel **a** (YEME10 + congocidine). The detailed genetic organization of the congocidine BGC is presented.

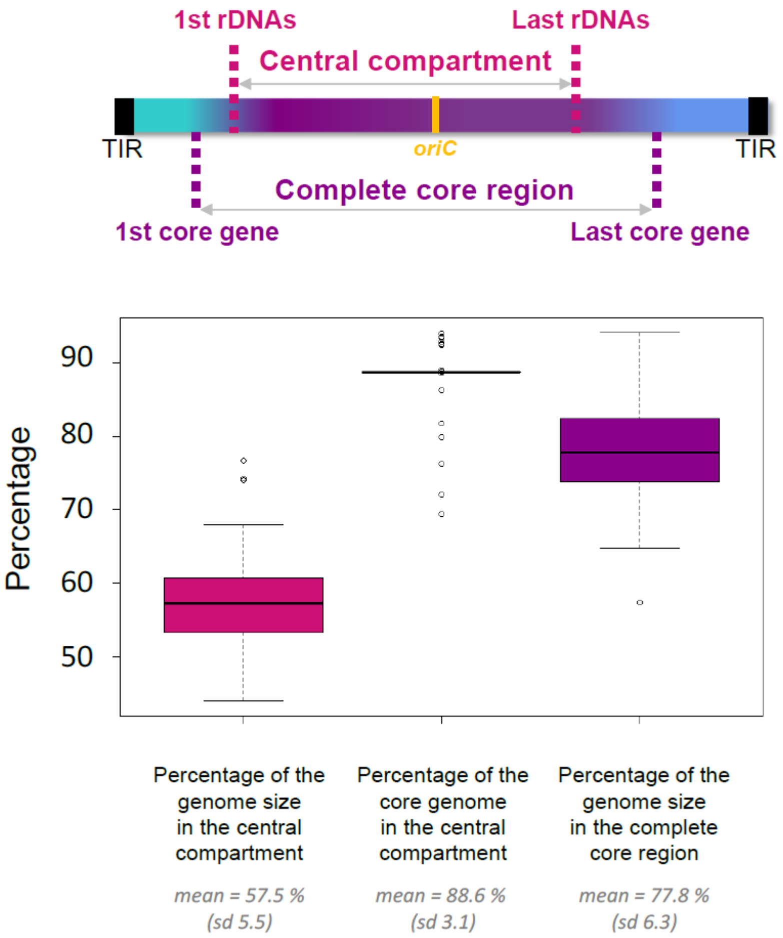

Supplementary Figure 8: Percentage of core genes within the central compartment in a panel of 125 *Streptomyces* genomes

The percentage of core CDSs located in the central region was assessed in the panel of 125 genomes representative of *Streptomyces* genus diversity. The sizes of the central compartment (delimitated by the first and last rDNA operons) and of the region encompassing 100 % of the core CDSs (‘complete core region’) were also determined and expressed as percentages relative to the size of the whole chromosome (plasmids were not considered). The boxplots represent the first quartile, median and third quartile. The upper whisker extends from the hinge to the largest value no further than 1.5 * the inter-quartile range (IQR, i.e. distance between the first and third quartiles) from the hinge. The lower whisker extends from the hinge to the smallest value at most 1.5 * IQR of the hinge. Outliers are represented (dots).

Supplementary table 1: Collection of 125 *Streptomyces* genomes used in this study

Name	Genome Assembly ID	Chromosome length (bp)	Plasmid length (bp)	CDSs nb	TIR length (bp)
Streptomyces sp. S1A1 7	ASM711356v2	11,713,151	292,353 [1]	11,068 [265]	ND
S. bingchenggensis BCW 1	ASM9238v1	11,936,683	-	10,216	ND
Streptomyces sp. 11 1 2	ASM359554v1	11,603,877	51,329 [1]	9,986 [61]	29,227
Streptomyces sp. GGCR 6	ASM395571v1	10,393,987	-	9,540	96,011
Streptomyces sp. CdTB01	ASM148456v1	9,902,731	288,836 [1]	9,444 [287]	400,291
Streptomyces sp. YIM 121038	ASM608871v1	10,130,554	967,890 [1]	9,440 [840]	128,054
S. hygroscopicus jinggangensis 5008	ASM24535v1	10,145,833	237,851 [2]	9,401 [243]	ND
Streptomyces sp. GY16	ASM918486v1	10,116,136	59,304 [1]	9,248 [66]	ND
Streptomyces sp. 769	ASM81602v1	10,100,774	237,512 [1]	9,180 [224]	155,968
Streptomyces sp. T44	ASM979628v1	9,735,779	-	9,126	ND
S. scabiei 87 22	ASM9130v1	10,148,695	-	9,009	18,488
Streptomyces sp. P3	ASM303247v1	9,851,971	-	8,820	ND
S. noursei ATCC 11455	ASM170427v1	9,815,884	-	8,811	170,209
S. kanamyceticus ATCC 12853	ASM870449v1	10,133,897	-	8,806	ND
S. chartreusis ATCC 14922	ASM870471v1	9,912,098	-	8,704	ND
S. atratus SCSIO ZH16	ASM333086v1	9,641,288	-	8,700	288,246
S. lincolnensis LC G	ASM334444v1	9,513,637	-	8,622	ND
S. griseorubiginosus 3E 1	ASM359523v1	9,512,378	-	8,551	113,251
S. lydicus A02	ASM95203v2	9,307,519	-	8,534	52,505
Streptomyces sp. CC0208	ASM344373v1	9,320,089	-	8,531	ND
S. coeruleorubidus ATCC 13740	ASM870513v1	9,335,698	-	8,518	ND
Streptomyces sp. NEAU S7GS2	ASM317327v1	9,641,634	45,805 [1]	8,508 [52]	ND
S. ectabilis ATCC 27465	ASM870479v1	9,807,160	-	8,329	112,273
S. coelicolor A3 2	ASM20383v1	8,667,507	387,340 [2]	8,320 [400]	21,653
S. formicae KY5	ASM255654v1	9,611,874	-	8,317	35,482
S. vietnamensis GIM4 0001	ASM83000v1	8,867,142	286,635 [1]	8,287 [271]	49,821
S. rimosus ATCC 10970	ASM870465v1	9,361,154	-	8,287	11,287
S. autolyticus CGMCC0516	ASM198397v1	10,029,028	155,632 [7]	8,279 [36]	ND
S. griseorubiginosus BTU6	ASM651693v1	9,226,027	-	8,274	224,191
S. albulus CK 15	ASM93518v3	9,336,218	-	8,241	ND
Streptomyces sp. S10 2016	ASM161179v1	9,083,372	-	8,241	134,209
S. dengpaensis XZHG99	ASM294683v1	8,541,354	168,817 [2]	8,236 [210]	21,003
Streptomyces sp. ADI95 16	ASM372149v1	8,184,000	902,421 [4]	8,223 [861]	ND
S. avermitilis MA 4680	ASM976v2	9,025,608	94,287 [1]	8,132 [94]	ND
S. lavendulae CCM 3239	ASM280384v1	8,691,711	241,081 [1]	8,085 [231]	237,734
S. chartreusis NRRL 3882	NRRL3882	8,983,317	-	8,083	39,831
S. ectabilis NRRL 2792	ASM736339v1	9,505,665	-	8,055	ND
Streptomyces sp. M2	ASM410450v1	8,718,751	-	8,025	ND
Streptomyces sp. SYP A7193	ASM949565v1	8,193,527	629,255 [2]	7,984 [571]	ND
S. lydicus WYEC 108	ASM399437v1	9,125,666	-	7,970	8,910
S. venezuelae ATCC 14584	ASM864231v1	8,942,078	-	7,935	165,025
S. lunaelactis MM109	ASM305455v1	8,396,100	174,091 [2]	7,873 [149]	ND
Streptomyces sp. Mg1	ASM41226v2	7,868,178	848,015 [3]	7,864 [812]	ND
Streptomyces sp. QMT 28	ASM949827v1	8,880,330	-	7,861	ND
S. griseoviridis F1 27	ASM399439v1	8,963,414	-	7,785	58,707
S. venezuelae ATCC 15068	ASM864237v1	8,558,202	-	7,743	ND
Streptomyces sp. Go 475	ASM333084v1	8,570,609	-	7,729	6,902
S. gilvosporeus F607	ASM208219v1	8,482,298	-	7,624	ND
S. olivoreticuli ATCC 31159	ASM339113v1	8,809,793	-	7,593	170,471
S. hundungensis BH38	ASM362781v1	8,393,044	-	7,541	65,122
S. ambofaciens ATCC 23877	ASM126788v1	8,303,940	89,658 [1]	7,541 [128]	202,695
S. alfalfae ACCC40021	ASM197502v1	8,625,867	-	7,525	149,241
Streptomyces sp. SUK 48	ASM965076v1	8,341,671	-	7,507	188,470
Streptomyces sp. CCM MD2014	ASM77204v1	8,274,043	-	7,501	14,382
S. pristinaespiralis HCCB 10218	ASM127807v1	8,532,592	-	7,498	419,828
S. platensis ATCC 23948	ASM870485v1	8,501,012	-	7,490	24,984
S. rochei 7434AN4	ASM806499v1	8,364,802	-	7,485	53,905
Streptomyces sp. W1SF4	ASM395003v1	7,272,878	795,265 [2]	7,420 [656]	ND
S. actuosus ATCC 25421	ASM320803v1	8,145,579	-	7,399	17,181
S. endophyte N2	ASM410448v1	8,428,700	-	7,398	53,557
Streptomyces sp. Sge12	ASM208045v1	7,983,613	127,085 [1]	7,376 [119]	162,199
S. pactum ACT12	ASM200522v1	8,550,793	-	7,375	239,610

Name: Species name
Genome Assembly ID: Unique identifier used in the NCBI Genome Assembly database
Chromosome length (bp): Chromosome length expressed in bp
Plasmid length (bp): Cumulative length of plasmids [Number of plasmids] expressed in bp
CDSs nb: Number of CDSs in chromosome [CDSs in plasmids]
TIR length (bp): Size of one copy of a TIR expressed in bp.

Supplementary table 1 (continued)

Name	Genome Assembly ID	Chromosome length (bp)	Plasmid length (bp)	CDSs nb	TIR length (bp)
Streptomyces sp. WAC 01438	ASM394552v1	8,138,328	62,839 [1]	7,350 [72]	49,769
S. lydicus GS93 23	ASM198444v1	8,243,179	-	7,320	36,373
S. griseus NBRC 13350	ASM1060v1	8,545,929	-	7,306	132,910
S. venezuelae ATCC 14583	ASM864235v1	8,018,484	-	7,301	157,265
Streptomyces sp. SS52	ASM479571v1	8,184,045	-	7,293	43,391
S. collinus Tu 365	ASM44487v1	8,272,925	104,361 [2]	7,283 [101]	631,364
Streptomyces sp. WAC 01529	ASM394554v1	8,270,461	-	7,269	58,922
Streptomyces sp. KPB2	ASM395005v1	8,082,236	-	7,256	40,616
S. ambofaciens DSM 40697	ASM163286v1	8,137,876	-	7,250	212,696
Streptomyces sp. fd1 xmd	ASM200768v1	7,929,999	-	7,236	ND
S. clavuligerus ATCC 27064 2 3	ASM551946v1	6,748,591	1,795,495 [1]	7,226 [1,534]	ND
Streptomyces sp. GSSD 12	ASM334496v1	8,454,852	-	7,201	ND
S. katrae S3	ASM202842v1	7,504,851	551,599 [2]	7,196 [321]	ND
Streptomyces sp. CFMR 7 CFMR 7	ASM127809v1	8,207,742	99,537 [1]	7,190 [95]	11,739
S. cattleya NRRL 8057	ASM23730v1	6,283,062	1,809,491 [1]	7,189 [1,651]	ND
S. tsukubensis AT3	ASM929602v1	8,615,214	18,766 [1]	7,183 [13]	17,935
S. venezuelae ATCC 14585	ASM864233v1	8,048,154	-	7,176	166,199
S. leeuwenhoekii sleC34	sleC34	7,903,895	218,596 [2]	7,120 [263]	388,272
Streptomyces sp. QHH 9511	ASM978963v1	7,524,079	91,197 [1]	7,109 [124]	ND
S. niveus SCSIO 3406	ASM200917v1	7,990,492	-	7,105	ND
Streptomyces sp. CB09001	ASM336979v1	7,787,608	-	7,085	ND
S. globisporus C 1027	ASM26134v2	7,608,611	174,988 [2]	7,079 [154]	ND
S. fulvissimus DSM 40593	ASM38594v1	7,905,758	-	7,072	ND
S. albireticuli MDJK11	ASM219245v1	8,144,417	-	7,024	133,288
S. vinaceus ATCC 27476	ASM870493v1	7,673,509	-	7,020	ND
S. venezuelae ATCC 10595	ASM870525v1	7,871,480	-	7,001	ND
S. galilaeus ATCC 14969	ASM870457v1	7,756,194	-	7,001	ND
Streptomyces sp. Tue6075	ASM193163v1	7,931,832	-	6,994	12,088
S. alboniger ATCC 12461	ASM870439v1	7,962,786	-	6,993	ND
S. nigra 452	ASM307405v1	7,641,029	-	6,992	107,944
S. violaceoruber S21	ASM208217v1	7,916,045	-	6,979	4,122
S. parvulus 2297	ASM166004v1	7,149,446	617,085 [1]	6,963 [443]	ND
S. bacillaris ATCC 15855	ASM326867v1	7,888,441	-	6,953	ND
Streptomyces sp. TN58	ASM194184v1	7,585,034	-	6,936	193,939
S. venezuelae ATCC 21018	ASM864227v1	7,746,267	-	6,919	ND
S. nodosus ATCC 14899 2	ASM870499v1	7,772,587	-	6,917	ND
S. albus ZD11	ASM367534v1	8,317,371	-	6,904	614,456
S. subrutilus ATCC 27467	ASM870453v1	7,604,974	-	6,839	162,000
S. fungicidicus TXX3120	ASM38594v1	6,740,768	926,728 [1]	6,839 [841]	ND
S. tsukubensis NRRL 18488	ASM393271v1	7,963,742	55,806 [2]	6,809 [63]	210,577
S. cinereoruber ATCC 19740	ASM929938v1	7,516,652	-	6,807	ND
S. prasinus ATCC 13879	ASM870444v1	7,647,592	-	6,793	ND
Streptomyces sp. SM18	ASM291077v2	7,703,166	-	6,783	14,611
Streptomyces sp. PAMC26508	ASM36480v1	7,526,197	104,048 [1]	6,779 [84]	36,502
S. nitrosporeus ATCC 12769	ASM870455v1	7,581,562	-	6,778	26,990
Streptomyces sp. SSL 25	ASM785615v1	8,146,484	-	6,768	132,197
S. glaucescens GLA O	ASM76121v1	7,453,200	170,574 [1]	6,715 [140]	14,128
S. asterosporus DSM 41452	ASM671613v1	7,766,581	-	6,710	ND
S. venezuelae ATCC 21782	ASM864229v1	7,525,322	-	6,699	182,221
S. cyaneogriseus noncyanogenus NMWT 1	ASM93144v1	7,762,396	-	6,681	ND
Streptomyces sp. SirexAA E	ASM17719v2	7,414,440	-	6,663	ND
Streptomyces sp. S8	ASM209499v1	7,529,075	72,789 [1]	6,624 [78]	12,184
S. viridosporus T7A ATCC 39115	ASM870451v1	7,280,536	-	6,603	ND
S. luteoverticillatus CGMCC 15060	ASM397071v1	7,367,863	-	6,574	ND
S. pluripotens MUSC 135	ASM80224v2	7,346,075	-	6,561	ND
Streptomyces sp. SAT1	ASM165449v1	7,472,530	-	6,503	ND
S. ficellus NRRL 8067	ASM973990v1	7,078,240	-	6,412	21,921
S. koyangensis VK A60T	ASM342892v1	7,220,839	-	6,365	20,855
S. tirandamycinicus HNM0039	ASM309751v1	7,289,495	-	6,363	ND
S. albidoflavus SM254	ASM157738v1	7,170,504	-	6,298	ND
S. ongiicola HNM0071	ASM312236v1	7,180,417	-	6,166	ND
S. fradiae ATCC 10745	ASM870442v1	6,725,579	-	5,877	48,424
S. seoulensis KCTC 9819	ASM432862v1	6,339,363	86,882 [1]	5,866 [107]	20,875

Name: Species name
Genome Assembly ID: Unique identifier used in the NCBI Genome Assembly database
Chromosome length (bp): Chromosome length expressed in bp
Plasmid length (bp): Cumulative length of plasmids [Number of plasmids] expressed in bp
CDSs nb: Number of CDSs in chromosome [CDSs in plasmids]
TIR length (bp): Size of one copy of a TIR expressed in bp

Supplementary table 2: Gene distribution in the central region *versus* the extremities (defined by the first and last rRNA operons) of *S. ambofaciens* ATCC23877 chromosome depending on features of interest

Features	Number of genes of interest	% of genes of interest present within the extremities	% of genes within the central region presenting the feature of interest	% of genes within the extremities presenting the feature of interest	Odds ratio[a]	*p*-value[b]
Chromosomal genes	7128	46.5				
Genome features related to the level of conservation						
CDSs of the actinobacterial signature	72	16.7	1.6	0.4	0.23	$1.4.10^{-7}$
Core CDSs	1020	11.3	23.7	3.5	0.12	$<2.2.10^{-16}$
CDSs with a high level of persistence (>0.95)	2186	17.6	47.2	11.6	0.15	$<2.2.10^{-16}$
GIs	1207	80.4	6.2	29.3	6.27	$<2.2.10^{-16}$
Strain-specific CDSs[c]	158	85.4	0.6	4.1	6.99	$<2.2.10^{-16}$
Genome features related to functional annotation						
Functional RNA (excluding rRNA)	75	13.3	1.7	0.3	0.17	$1.3.10^{-9}$
CDSs encoding functions involved in translation	175	13.7	4.0	0.7	0.18	$<2.2.10^{-16}$
NAPSF genes	49	26.5	0.9	0.4	0.41	0.005798
SMBGCs	385	76.4	2.4	8.9	3.98	$<2.2.10^{-16}$
Features related to the level of expression						
Genes always expressed at very high level (CAT_4)[d]	332	11.7	7.7	1.2	0.14	$<2.2.10^{-16}$
Genes always expressed at very low level (CAT_0)[d]	849	72.3	6.2	18.5	3.46	$<2.2.10^{-16}$

[a]The odds ratio represents the odds than an outcome will occur within the extremities compared to the odds of the outcome occurring in the central region. The genetic information of each TIR was taken into consideration for the calculations.
[b]The statistical significance of the odd ratios was assessed by two-sided Fisher's exact tests for count data.
[c]For this calculation, only the chromosomal strain-specific CDSs were taken into account (excluding the 44 strain-specific CDSs present on pSAM1 non-integrative plasmid).
[d]'always' refers to all the conditions investigated in this study (i.e. C1 to C7). Only chromosomal genes were considered in the calculation.

Supplementary References

1. Wang, X. *et al.* In Vivo Evidence for ATPase-Dependent DNA Translocation by the *Bacillus subtilis* SMC Condensin Complex. *Mol Cell* **71**, 841-847.e5 (2018).
2. Wang, X., Brandão, H. B., Le, T. B. K., Laub, M. T. & Rudner, D. Z. *Bacillus subtilis* SMC complexes juxtapose chromosome arms as they travel from origin to terminus. *Science* **355**, 524–527 (2017).
3. Lesne, A., Riposo, J., Roger, P., Cournac, A. & Mozziconacci, J. 3D genome reconstruction from chromosomal contacts. *Nat Methods* **11**, 1141–1143 (2014).
