# Supplementary material for: Dynamics of the compartmentalized Streptomyces chromosome during metabolic differentiation
Source: Nat Commun. 2021 Sep 1;12:5221. doi: 10.1038/s41467-021-25462-1 (PMC8410849; doi:10.1038/s41467-021-25462-1)
Supplement: Supplementary file 3 — Description of Additional Supplementary Files [file 41467_2021_25462_MOESM3_ESM.pdf]

Description of Additional Supplementary Files

File Name : Supplementary Data 1

Description : List of the GIs identified in Streptomyces ambofaciens ATCC 23877 genome

File Name : Supplementary Data 2

Description : List of the SMBGCs identified in Streptomyces ambofaciens ATCC 23877 genome

File Name: Supplementary Data 3

Description: Overall results from the comparative genomics, RNA-seq and 3C-seq analyses. The legend and the data are available in the 'Readme_Legend' and 'DATA' datasheets, respectively. We propose to add a Supplementary Data presenting the statistical report of the RNA-seq analysis (generated by SARTools R Packge). This information will be cited in the Method section and in the Report summary (to explicite our quality control approach). This file also contains the information about the version of the software that were used for the RNA-seq analysis

File Name: Supplementary Data 4

Description: Statistical report of the RNA-seq analysis (pairwise comparisons of conditions with DESeq2)
