# Supplementary material for: Dynamics of the compartmentalized Streptomyces chromosome during metabolic differentiation
Source: Nat Commun. 2021 Sep 1;12:5221. doi: 10.1038/s41467-021-25462-1 (PMC8410849; doi:10.1038/s41467-021-25462-1)
Supplement: Supplementary file 7 — Supplementary Data 4 [file 41467_2021_25462_MOESM7_ESM.zip › Supplementary Data 4_Nature_Communications.html]

 

 

 

 
 
 


 

 

 Statistical report of project Streptomics: pairwise comparison(s) of conditions with DESeq2 

 
 
 
 
 
 
 
 
 
 
 
 

 
 
 


 


 

 

 


 

 
 

 


 

 

 
 
 
 
 
 

 


 


 Statistical report of project Streptomics: pairwise comparison(s) of conditions with DESeq2 
  Stéphanie Bury-Moné  
  2020-08-27  

 


 The SARTools R package which generated this report has been developped at PF2 - Institut Pasteur by M.-A. Dillies and H. Varet ( hugo.varet@pasteur.fr ). Thanks to cite H. Varet, L. Brillet-Guéguen, J.-Y. Coppee and M.-A. Dillies,  SARTools: A DESeq2- and EdgeR-Based R Pipeline for Comprehensive Differential Analysis of RNA-Seq Data , PLoS One, 2016, doi:  http://dx.doi.org/10.1371/journal.pone.0157022  when using this tool for any analysis published. 
 
  1  Introduction 
 The analyses reported in this document are part of the Streptomics project. The aim is to find features that are differentially expressed between C1, C2, C3, C4, C5, C6 and C7. The statistical analysis process includes data normalization, graphical exploration of raw and normalized data, test for differential expression for each feature between the conditions, raw p-value adjustment and export of lists of features having a significant differential expression between the conditions. 
 The analysis is performed using the R software  [1] , Bioconductor  [2]  packages including DESeq2  [3,4]  and the SARTools package developed at PF2 - Institut Pasteur. Normalization and differential analysis are carried out according to the DESeq2 model and package. This report comes with additional tab-delimited text files that contain lists of differentially expressed features. 
 For more details about the DESeq2 methodology, please refer to its related publications  [3,4] . 
 
 
  2  Description of raw data 
 The count data files and associated biological conditions are listed in the following table. 
 
 
 
 
Table 1: Data files and associated biological conditions.
 
 
 
Name
 
 
File
 
 
Condition
 
 
Batch
 
 
 
 
C1-a
 
 
MP5-24-1_counts_S.txt
 
 
C1
 
 
RNA3-1
 
 
 
 
C1-b
 
 
MP5-24-2_counts_S.txt
 
 
C1
 
 
RNA3-2
 
 
 
 
C1-c
 
 
MP5-24-3_counts_S.txt
 
 
C1
 
 
RNA3-3
 
 
 
 
C2-a
 
 
MP5-30-1_counts_S.txt
 
 
C2
 
 
RNA3-1
 
 
 
 
C2-b
 
 
MP5-30-2_counts_S.txt
 
 
C2
 
 
RNA3-2
 
 
 
 
C2-c
 
 
MP5-30-3_counts_S.txt
 
 
C2
 
 
RNA3-3
 
 
 
 
C3-a
 
 
MP5-36-1_counts_S.txt
 
 
C3
 
 
RNA3-1
 
 
 
 
C3-b
 
 
MP5-36-2_counts_S.txt
 
 
C3
 
 
RNA3-2
 
 
 
 
C4-a
 
 
MP5-48-1_counts_S.txt
 
 
C4
 
 
RNA3-1
 
 
 
 
C4-b
 
 
MP5-48-2_counts_S.txt
 
 
C4
 
 
RNA3-2
 
 
 
 
C4-c
 
 
MP5-48-3_counts_S.txt
 
 
C4
 
 
RNA3-3
 
 
 
 
C5-a
 
 
WTMP5-72-1_counts_S.txt
 
 
C5
 
 
RNA2-9
 
 
 
 
C5-b
 
 
WTMP5-72-2_counts_S.txt
 
 
C5
 
 
RNA2-10
 
 
 
 
C5-c
 
 
WTMP5-72-3_counts_S.txt
 
 
C5
 
 
RNA2-11
 
 
 
 
C5-d
 
 
WTMP5-72-4_counts_S.txt
 
 
C5
 
 
RNA2-10
 
 
 
 
C6-a
 
 
Y0-2_counts_S.txt
 
 
C6
 
 
RNA3-1
 
 
 
 
C6-b
 
 
Y0-3_counts_S.txt
 
 
C6
 
 
RNA3-2
 
 
 
 
C6-c
 
 
Y0-4_counts_S.txt
 
 
C6
 
 
RNA3-3
 
 
 
 
C7-a
 
 
Y48-1_counts_S.txt
 
 
C7
 
 
RNA3-1
 
 
 
 
C7-b
 
 
Y48-2_counts_S.txt
 
 
C7
 
 
RNA3-2
 
 
 
 
C7-c
 
 
Y48-3_counts_S.txt
 
 
C7
 
 
RNA3-3
 
 
 
 After loading the data we first have a look at the raw data table itself. The data table contains one row per annotated feature and one column per sequenced sample. Row names of this table are feature IDs (unique identifiers). The table contains raw count values representing the number of reads that map onto the features. For this project, there are 7275 features in the count data table. 
 
 
 
 
Table 2: Partial view of the count data table.
 
 
 
 
 
C1-a
 
 
C1-b
 
 
C1-c
 
 
C2-a
 
 
C2-b
 
 
C2-c
 
 
C3-a
 
 
C3-b
 
 
C4-a
 
 
C4-b
 
 
C4-c
 
 
C5-a
 
 
C5-b
 
 
C5-c
 
 
C5-d
 
 
C6-a
 
 
C6-b
 
 
C6-c
 
 
C7-a
 
 
C7-b
 
 
C7-c
 
 
 
 
gene-SAM23877_RS00010
 
 
19
 
 
9
 
 
18
 
 
8
 
 
9
 
 
50
 
 
65
 
 
14
 
 
7
 
 
7
 
 
19
 
 
137
 
 
36
 
 
21
 
 
23
 
 
8
 
 
24
 
 
12
 
 
48
 
 
115
 
 
35
 
 
 
 
gene-SAM23877_RS00015
 
 
14
 
 
5
 
 
20
 
 
16
 
 
2
 
 
65
 
 
71
 
 
13
 
 
4
 
 
11
 
 
35
 
 
69
 
 
29
 
 
17
 
 
17
 
 
5
 
 
39
 
 
4
 
 
64
 
 
133
 
 
51
 
 
 
 
gene-SAM23877_RS00020
 
 
460
 
 
340
 
 
249
 
 
185
 
 
142
 
 
163
 
 
174
 
 
68
 
 
420
 
 
447
 
 
263
 
 
143
 
 
163
 
 
68
 
 
118
 
 
293
 
 
413
 
 
199
 
 
351
 
 
517
 
 
487
 
 
 
 
gene-SAM23877_RS00030
 
 
173
 
 
162
 
 
121
 
 
293
 
 
171
 
 
389
 
 
4226
 
 
1322
 
 
27166
 
 
24736
 
 
8740
 
 
1798
 
 
2494
 
 
1704
 
 
2169
 
 
163
 
 
254
 
 
55
 
 
3719
 
 
6591
 
 
5138
 
 
 
 
gene-SAM23877_RS00035
 
 
1
 
 
0
 
 
0
 
 
0
 
 
0
 
 
2
 
 
2
 
 
0
 
 
9
 
 
5
 
 
2
 
 
5
 
 
17
 
 
3
 
 
19
 
 
1
 
 
0
 
 
1
 
 
4
 
 
2
 
 
3
 
 
 
 
gene-SAM23877_RS00050
 
 
247
 
 
190
 
 
105
 
 
84
 
 
66
 
 
54
 
 
43
 
 
30
 
 
103
 
 
83
 
 
56
 
 
29
 
 
33
 
 
13
 
 
41
 
 
217
 
 
208
 
 
50
 
 
46
 
 
43
 
 
37
 
 
 
 Looking at the summary of the count table provides a basic description of these raw counts (min and max values, median, etc). 
 
 
 
 
Table 3: Summary of the raw counts.
 
 
 
 
 
C1-a
 
 
C1-b
 
 
C1-c
 
 
C2-a
 
 
C2-b
 
 
C2-c
 
 
C3-a
 
 
C3-b
 
 
C4-a
 
 
C4-b
 
 
C4-c
 
 
C5-a
 
 
C5-b
 
 
C5-c
 
 
C5-d
 
 
C6-a
 
 
C6-b
 
 
C6-c
 
 
C7-a
 
 
C7-b
 
 
C7-c
 
 
 
 
Min.
 
 
0
 
 
0
 
 
0
 
 
0
 
 
0
 
 
0
 
 
0
 
 
0
 
 
0
 
 
0
 
 
0
 
 
0
 
 
0
 
 
0
 
 
0
 
 
0
 
 
0
 
 
0
 
 
0
 
 
0
 
 
0
 
 
 
 
1st Qu.
 
 
49
 
 
41
 
 
37
 
 
26
 
 
23
 
 
35
 
 
29
 
 
17
 
 
49
 
 
53
 
 
55
 
 
12
 
 
17
 
 
11
 
 
12
 
 
29
 
 
40
 
 
19
 
 
40
 
 
60
 
 
52
 
 
 
 
Median
 
 
311
 
 
261
 
 
218
 
 
150
 
 
132
 
 
188
 
 
175
 
 
97
 
 
224
 
 
251
 
 
276
 
 
57
 
 
84
 
 
57
 
 
59
 
 
202
 
 
257
 
 
113
 
 
187
 
 
284
 
 
241
 
 
 
 
Mean
 
 
3640
 
 
3205
 
 
2415
 
 
2049
 
 
2406
 
 
2237
 
 
2049
 
 
1593
 
 
2616
 
 
2654
 
 
2248
 
 
1438
 
 
1151
 
 
1012
 
 
1076
 
 
2610
 
 
2557
 
 
3287
 
 
1659
 
 
2303
 
 
2282
 
 
 
 
3rd Qu.
 
 
1599
 
 
1412
 
 
1014
 
 
731
 
 
621
 
 
766
 
 
761
 
 
413
 
 
1023
 
 
1075
 
 
1183
 
 
265
 
 
407
 
 
275
 
 
307
 
 
1153
 
 
1319
 
 
566
 
 
771
 
 
1139
 
 
1000
 
 
 
 
Max.
 
 
3026493
 
 
3032889
 
 
4092460
 
 
5100698
 
 
8990875
 
 
6811892
 
 
6451389
 
 
6159413
 
 
3345911
 
 
5619239
 
 
2763745
 
 
6053085
 
 
2048044
 
 
2791157
 
 
2391632
 
 
3106591
 
 
2470348
 
 
12537098
 
 
2315830
 
 
3463676
 
 
2938192
 
 
 
 Figure 1 shows the total number of mapped and counted reads for each sample. We expect total read counts to be similar within conditions, they may be different across conditions. Total counts sometimes vary widely between replicates. This may happen for several reasons, including: 
 
 different rRNA contamination levels between samples (even between biological replicates); 
 slight differences between library concentrations, since they may be difficult to measure with high precision. 
 
 
 
 
 Figure 1: Number of mapped reads per sample. Colors refer to the biological condition of the sample. 
 
 
 Figure 2 shows the percentage of features with no read count in each sample. We expect this percentage to be similar within conditions. Features with null read counts in the 21 samples are left in the data but are not taken into account for the analysis with DESeq2. Here, 187 features (2.57%) are in this situation (dashed line). Results for those features (fold-change and p-values) are set to NA in the results files. 
 
 
 
 Figure 2: Percentage of features with null read counts in each sample. 
 
 
 Figure 3 shows the distribution of read counts for each sample. For sake of readability,  \(\text{log}_2(\text{counts}+1)\)  are used instead of raw counts. Again we expect replicates to have similar distributions. In addition, this figure shows if read counts are preferably low, medium or high. This depends on the organisms as well as the biological conditions under consideration. 
 
 
 
 Figure 3: Density distribution of read counts. 
 
 
 It may happen that one or a few features capture a high proportion of reads (up to 20% or more). This phenomenon should not influence the normalization process. The DESeq2 normalization has proved to be robust to this situation [Dillies, 2012]. Anyway, we expect these high count features to be the same across replicates. They are not necessarily the same across conditions. Figure 4 and table 4 illustrate the possible presence of such high count features in the data set. 
 
 
 
 Figure 4: Percentage of reads associated with the sequence having the highest count (provided in each box on the graph) for each sample. 
 
 
 
 
 
 
Table 4: Percentage of reads associated with the sequences having the highest counts.
 
 
 
 
 
C1-a
 
 
C1-b
 
 
C1-c
 
 
C2-a
 
 
C2-b
 
 
C2-c
 
 
C3-a
 
 
C3-b
 
 
C4-a
 
 
C4-b
 
 
C4-c
 
 
C5-a
 
 
C5-b
 
 
C5-c
 
 
C5-d
 
 
C6-a
 
 
C6-b
 
 
C6-c
 
 
C7-a
 
 
C7-b
 
 
C7-c
 
 
 
 
gene-SAM23877_RS36805
 
 
11.43
 
 
13.01
 
 
23.29
 
 
34.22
 
 
51.36
 
 
41.85
 
 
43.27
 
 
53.16
 
 
17.58
 
 
29.10
 
 
16.90
 
 
57.88
 
 
24.47
 
 
37.92
 
 
30.55
 
 
16.36
 
 
13.28
 
 
52.42
 
 
19.18
 
 
20.67
 
 
17.70
 
 
 
 
gene-SAM23877_RS36150
 
 
2.46
 
 
3.49
 
 
5.00
 
 
8.84
 
 
11.28
 
 
8.55
 
 
4.10
 
 
9.35
 
 
1.11
 
 
1.88
 
 
1.80
 
 
7.81
 
 
2.15
 
 
3.41
 
 
2.32
 
 
3.97
 
 
4.35
 
 
14.07
 
 
2.91
 
 
2.74
 
 
3.13
 
 
 
 
gene-SAM23877_RS09750
 
 
1.55
 
 
1.43
 
 
0.89
 
 
0.79
 
 
0.46
 
 
0.52
 
 
0.40
 
 
0.27
 
 
0.42
 
 
0.40
 
 
0.49
 
 
0.22
 
 
0.54
 
 
0.48
 
 
0.38
 
 
1.39
 
 
1.26
 
 
0.31
 
 
0.96
 
 
0.96
 
 
0.91
 
 
 
 
gene-SAM23877_RS21195
 
 
1.29
 
 
1.16
 
 
0.96
 
 
0.67
 
 
0.32
 
 
0.35
 
 
0.19
 
 
0.11
 
 
0.15
 
 
0.10
 
 
0.22
 
 
0.02
 
 
0.05
 
 
0.04
 
 
0.04
 
 
0.99
 
 
0.97
 
 
0.42
 
 
0.03
 
 
0.03
 
 
0.03
 
 
 
 
gene-SAM23877_RS20495
 
 
0.74
 
 
0.72
 
 
0.95
 
 
1.03
 
 
0.97
 
 
0.46
 
 
0.49
 
 
0.29
 
 
0.26
 
 
0.35
 
 
0.39
 
 
0.07
 
 
0.08
 
 
0.09
 
 
0.06
 
 
0.75
 
 
1.19
 
 
0.66
 
 
0.23
 
 
0.29
 
 
0.18
 
 
 
 
gene-SAM23877_RS21615
 
 
0.57
 
 
0.49
 
 
0.48
 
 
0.31
 
 
0.20
 
 
0.39
 
 
0.55
 
 
0.26
 
 
0.03
 
 
0.03
 
 
0.25
 
 
0.10
 
 
0.20
 
 
0.18
 
 
0.24
 
 
0.48
 
 
0.47
 
 
0.35
 
 
0.04
 
 
0.04
 
 
0.04
 
 
 
 
gene-SAM23877_RS11090
 
 
0.00
 
 
0.00
 
 
0.00
 
 
0.00
 
 
0.00
 
 
0.00
 
 
0.26
 
 
0.56
 
 
0.02
 
 
0.06
 
 
0.28
 
 
0.00
 
 
0.00
 
 
0.00
 
 
0.00
 
 
0.00
 
 
0.00
 
 
0.00
 
 
0.00
 
 
0.00
 
 
0.00
 
 
 
 
gene-SAM23877_RS15400
 
 
0.02
 
 
0.02
 
 
0.01
 
 
0.08
 
 
0.02
 
 
0.00
 
 
0.08
 
 
0.05
 
 
1.27
 
 
0.63
 
 
0.12
 
 
0.16
 
 
0.78
 
 
0.60
 
 
0.91
 
 
0.03
 
 
0.01
 
 
0.00
 
 
0.31
 
 
0.27
 
 
0.39
 
 
 
 
gene-SAM23877_RS15410
 
 
0.02
 
 
0.02
 
 
0.01
 
 
0.11
 
 
0.04
 
 
0.00
 
 
0.14
 
 
0.09
 
 
1.12
 
 
0.59
 
 
0.12
 
 
0.07
 
 
0.27
 
 
0.24
 
 
0.29
 
 
0.03
 
 
0.02
 
 
0.01
 
 
0.35
 
 
0.33
 
 
0.43
 
 
 
 
gene-SAM23877_RS22575
 
 
0.02
 
 
0.09
 
 
0.06
 
 
0.09
 
 
0.04
 
 
0.06
 
 
0.16
 
 
0.20
 
 
0.77
 
 
0.46
 
 
1.02
 
 
0.05
 
 
0.17
 
 
0.11
 
 
0.23
 
 
0.05
 
 
0.04
 
 
0.00
 
 
0.09
 
 
0.09
 
 
0.09
 
 
 
 
gene-SAM23877_RS11470
 
 
0.08
 
 
0.10
 
 
0.10
 
 
0.10
 
 
0.05
 
 
0.06
 
 
0.13
 
 
0.08
 
 
0.36
 
 
0.33
 
 
0.16
 
 
0.66
 
 
1.73
 
 
1.27
 
 
2.02
 
 
0.09
 
 
0.12
 
 
0.03
 
 
0.40
 
 
0.38
 
 
0.42
 
 
 
 
gene-SAM23877_RS18505
 
 
0.65
 
 
0.32
 
 
0.31
 
 
0.11
 
 
0.13
 
 
0.17
 
 
0.14
 
 
0.07
 
 
0.09
 
 
0.12
 
 
0.08
 
 
0.13
 
 
0.38
 
 
0.27
 
 
0.33
 
 
0.35
 
 
0.37
 
 
0.71
 
 
0.10
 
 
0.10
 
 
0.07
 
 
 
 
gene-SAM23877_RS03010
 
 
0.00
 
 
0.00
 
 
0.00
 
 
0.00
 
 
0.00
 
 
0.00
 
 
0.00
 
 
0.00
 
 
0.01
 
 
0.01
 
 
0.00
 
 
0.00
 
 
0.00
 
 
0.00
 
 
0.00
 
 
0.00
 
 
0.00
 
 
0.00
 
 
0.86
 
 
0.69
 
 
0.98
 
 
 
 We may wish to assess the similarity between samples across conditions. A pairwise scatter plot is produced (figure 5) to show how replicates and samples from different biological conditions are similar or different ( \(\text{log}_2(\text{counts}+1)\)  are used instead of raw count values). Moreover, as the Pearson correlation has been shown not to be relevant to measure the similarity between replicates, the SERE statistic has been proposed as a similarity index between RNA-Seq samples  [5] . It measures whether the variability between samples is random Poisson variability or higher. Pairwise SERE values are printed in the lower triangle of the pairwise scatter plot. The value of the SERE statistic is: 
 
  0 when samples are identical (no variability at all: this may happen in the case of a sample duplication);  
  1 for technical replicates (technical variability follows a Poisson distribution);  
  greater than 1 for biological replicates and samples from different biological conditions (biological variability is higher than technical one, data are over-dispersed with respect to Poisson). The higher the SERE value, the lower the similarity. It is expected to be lower between biological replicates than between samples of different biological conditions. Hence, the SERE statistic can be used to detect inversions between samples.  
 
 
 
 
 Figure 5: Pairwise comparison of samples (not produced when more than 30 samples). 
 
 
 
 
  3  Variability within the experiment: data exploration 
 The main variability within the experiment is expected to come from biological differences between the samples. This can be checked in two ways. The first one is to perform a hierarchical clustering of the whole sample set. This is performed after a transformation of the count data which can be either a Variance Stabilizing Transformation (VST) or a regularized log transformation (rlog)  [3,4] . 
 A VST is a transformation of the data that makes them homoscedastic, meaning that the variance is then independent of the mean. It is performed in two steps: (i) a mean-variance relationship is estimated from the data with the same function that is used to normalize count data and (ii) from this relationship, a transformation of the data is performed in order to get a dataset in which the variance is independent of the mean. The homoscedasticity is a prerequisite for the use of some data analysis methods, such as hierarchical clustering or Principal Component Analysis (PCA). The regularized log transformation is based on a GLM (Generalized Linear Model) on the counts and has the same goal as a VST but is more robust in the case when the size factors vary widely. 
 Figure 6 shows the dendrogram obtained from VST-transformed data. An euclidean distance is computed between samples, and the dendrogram is built upon the Ward criterion. We expect this dendrogram to group replicates and separate biological conditions. 
 
 
 
 Figure 6: Sample clustering based on normalized data. 
 
 
 Another way of visualizing the experiment variability is to look at the first principal components of the PCA, as shown on the figure 7. On this figure, the first principal component (PC1) is expected to separate samples from the different biological conditions, meaning that the biological variability is the main source of variance in the data. 
 
 
 
 Figure 7: First two components of a Principal Component Analysis, with percentages of variance associated with each axis. 
 
 
 
 
  4  Normalization 
 Normalization aims at correcting systematic technical biases in the data, in order to make read counts comparable across samples. The normalization proposed by DESeq2 relies on the hypothesis that most features are not differentially expressed. It computes a scaling factor for each sample. Normalized read counts are obtained by dividing raw read counts by the scaling factor associated with the sample they belong to. Scaling factors around 1 mean (almost) no normalization is performed. Scaling factors lower than 1 will produce normalized counts higher than raw ones, and the other way around. Two options are available to compute scaling factors: locfunc=“median” (default) or locfunc=“shorth”. Here, the normalization was performed with locfunc=“median”. 
 
 
 
 
Table 5: Normalization factors.
 
 
 
 
 
1
 
 
2
 
 
3
 
 
4
 
 
5
 
 
6
 
 
7
 
 
8
 
 
9
 
 
10
 
 
11
 
 
12
 
 
13
 
 
14
 
 
15
 
 
16
 
 
17
 
 
18
 
 
19
 
 
20
 
 
21
 
 
 
 
Size factor
 
 
2.21
 
 
1.90
 
 
1.45
 
 
0.99
 
 
0.83
 
 
1.10
 
 
0.98
 
 
0.51
 
 
1.37
 
 
1.42
 
 
1.49
 
 
0.34
 
 
0.52
 
 
0.35
 
 
0.38
 
 
1.44
 
 
1.79
 
 
0.76
 
 
1.08
 
 
1.58
 
 
1.41
 
 
 
 The histograms (figure 8) can help to validate the choice of the normalization parameter (“median” or “shorth”). Under the hypothesis that most features are not differentially expressed, each size factor represented by a red line is expected to be close to the mode of the distribution of the counts divided by their geometric means across samples. 
 
 
 
 Figure 8: Diagnostic of the estimation of the size factors. 
 
 
 The figure 9 shows that the scaling factors of DESeq2 and the total count normalization factors may not perform similarly. 
 
 
 
 Figure 9: Plot of the estimated size factors and the total number of reads per sample. 
 
 
 Boxplots are often used as a qualitative measure of the quality of the normalization process, as they show how distributions are globally affected during this process. We expect normalization to stabilize distributions across samples. Figure 10 shows boxplots of raw (left) and normalized (right) data respectively. 
 
 
 
 Figure 10: Boxplots of raw (left) and normalized (right) read counts. 
 
 
 
 
  5  Differential analysis 
 
  5.1  Modelisation 
 DESeq2 aims at fitting one linear model per feature. For this project, the design used is counts ~ Condition and the goal is to estimate the models’ coefficients which can be interpreted as  \(\log_2(\texttt{FC})\) . These coefficients will then be tested to get p-values and adjusted p-values. 
 
 
  5.2  Outlier detection 
 Model outliers are features for which at least one sample seems unrelated to the experimental or study design. For every feature and for every sample, the Cook’s distance  [6]  reflects how the sample matches the model. A large value of the Cook’s distance indicates an outlier count and p-values are not computed for the corresponding feature. 
 
 
  5.3  Dispersions estimation 
 The DESeq2 model assumes that the count data follow a negative binomial distribution which is a robust alternative to the Poisson law when data are over-dispersed (the variance is higher than the mean). The first step of the statistical procedure is to estimate the dispersion of the data. Its purpose is to determine the shape of the mean-variance relationship. The default is to apply a GLM (Generalized Linear Model) based method (fitType=“parametric”), which can handle complex designs but may not converge in some cases. The alternative is to use fitType=“local” as described in the original paper  [3]  or fitType=“mean”. The parameter used for this project is fitType=“parametric”. Then, DESeq2 imposes a Cox Reid-adjusted profile likelihood maximization  [7]  and uses the maximum  a posteriori  (MAP) of the dispersion [Wu, 2013]. 
 
 
 
 Figure 11: Dispersion estimates (left) and diagnostic of log-normality (right). 
 
 
 The left panel on figure 11 shows the result of the dispersion estimation step. The x- and y-axes represent the mean count value and the estimated dispersion respectively. Black dots represent empirical dispersion estimates for each feature (from the observed counts). The red dots show the mean-variance relationship function (fitted dispersion value) as estimated by the model. The blue dots are the final estimates from the maximum  a posteriori  and are used to perform the statistical test. Blue circles (if any) point out dispersion outliers. These are features with a very high empirical variance (computed from observed counts). These high dispersion values fall far from the model estimation. For these features, the statistical test is based on the empirical variance in order to be more conservative than with the MAP dispersion. These features will have low chance to be declared significant. The figure on the right panel allows to check the hypothesis of log-normality of the dispersions. 
 
 
  5.4  Statistical test for differential expression 
 Once the dispersion estimation and the model fitting have been done, DESeq2 can perform the statistical testing. Figure 12 shows the distributions of raw p-values computed by the statistical test for the comparison(s) done. This distribution is expected to be a mixture of a uniform distribution on  \([0,1]\)  and a peak around 0 corresponding to the differentially expressed features. 
 
 
 
 Figure 12: Distribution(s) of raw p-values. 
 
 
 
 
  5.5  Independent filtering 
 DESeq2 can perform an independent filtering to increase the detection power of differentially expressed features at the same experiment-wide type I error. Since features with very low counts are not likely to see significant differences typically due to high dispersion, it defines a threshold on the mean of the normalized counts irrespective of the biological condition. This procedure is independent because the information about the variables in the design formula is not used  [4] . 
Table 6 reports the thresholds used for each comparison and the number of features discarded by the independent filtering. Adjusted p-values of discarded features are then set to NA.    
 
 
Table 6: Number of features discarded by the independent filtering for each comparison.
 
 
 
Test vs Ref
 
 
Threshold
 
 
# discarded
 
 
 
 
C2 vs C1
 
 
2.54
 
 
325
 
 
 
 
C3 vs C1
 
 
0.03
 
 
187
 
 
 
 
C4 vs C1
 
 
0.03
 
 
187
 
 
 
 
C5 vs C1
 
 
0.03
 
 
187
 
 
 
 
C6 vs C1
 
 
7.15
 
 
599
 
 
 
 
C7 vs C1
 
 
0.03
 
 
187
 
 
 
 
C3 vs C2
 
 
2.54
 
 
325
 
 
 
 
C4 vs C2
 
 
0.03
 
 
187
 
 
 
 
C5 vs C2
 
 
0.03
 
 
187
 
 
 
 
C6 vs C2
 
 
7.15
 
 
599
 
 
 
 
C7 vs C2
 
 
0.03
 
 
187
 
 
 
 
C4 vs C3
 
 
4.9
 
 
462
 
 
 
 
C5 vs C3
 
 
0.03
 
 
187
 
 
 
 
C6 vs C3
 
 
0.03
 
 
187
 
 
 
 
C7 vs C3
 
 
0.03
 
 
187
 
 
 
 
C5 vs C4
 
 
0.03
 
 
187
 
 
 
 
C6 vs C4
 
 
0.03
 
 
187
 
 
 
 
C7 vs C4
 
 
0.03
 
 
187
 
 
 
 
C6 vs C5
 
 
0.03
 
 
187
 
 
 
 
C7 vs C5
 
 
0.03
 
 
187
 
 
 
 
C7 vs C6
 
 
0.03
 
 
187
 
 
 
 
 
  5.6  Final results 
 A p-value adjustment is performed to take into account multiple testing and control the false positive rate to a chosen level  \(\alpha\) . For this analysis, a BH p-value adjustment was performed  [8]  and the level of controlled false positive rate was set to 0.05. 
 
 
 
 
Table 7: Number of up-, down- and total number of differentially expressed features for each comparison.
 
 
 
Test vs Ref
 
 
# down
 
 
# up
 
 
# total
 
 
 
 
C2 vs C1
 
 
830
 
 
1014
 
 
1844
 
 
 
 
C3 vs C1
 
 
1659
 
 
1614
 
 
3273
 
 
 
 
C4 vs C1
 
 
2090
 
 
2208
 
 
4298
 
 
 
 
C5 vs C1
 
 
2425
 
 
2577
 
 
5002
 
 
 
 
C6 vs C1
 
 
464
 
 
532
 
 
996
 
 
 
 
C7 vs C1
 
 
2404
 
 
2560
 
 
4964
 
 
 
 
C3 vs C2
 
 
757
 
 
897
 
 
1654
 
 
 
 
C4 vs C2
 
 
1810
 
 
1999
 
 
3809
 
 
 
 
C5 vs C2
 
 
2252
 
 
2342
 
 
4594
 
 
 
 
C6 vs C2
 
 
784
 
 
750
 
 
1534
 
 
 
 
C7 vs C2
 
 
2207
 
 
2265
 
 
4472
 
 
 
 
C4 vs C3
 
 
835
 
 
1129
 
 
1964
 
 
 
 
C5 vs C3
 
 
1875
 
 
1910
 
 
3785
 
 
 
 
C6 vs C3
 
 
1482
 
 
1781
 
 
3263
 
 
 
 
C7 vs C3
 
 
1918
 
 
1871
 
 
3789
 
 
 
 
C5 vs C4
 
 
2071
 
 
1814
 
 
3885
 
 
 
 
C6 vs C4
 
 
2208
 
 
2236
 
 
4444
 
 
 
 
C7 vs C4
 
 
1921
 
 
1680
 
 
3601
 
 
 
 
C6 vs C5
 
 
2502
 
 
2418
 
 
4920
 
 
 
 
C7 vs C5
 
 
1984
 
 
1927
 
 
3911
 
 
 
 
C7 vs C6
 
 
2437
 
 
2480
 
 
4917
 
 
 
 Figure 13 represents the MA-plot of the data for the comparisons done, where differentially expressed features are highlighted in red. A MA-plot represents the log ratio of differential expression as a function of the mean intensity for each feature. Triangles correspond to features having a too low/high  \(\log_2(\text{FC})\)  to be displayed on the plot. 
 
 
 
 Figure 13: MA-plot(s) of each comparison. Red dots represent significantly differentially expressed features. 
 
 
 Figure 14 shows the volcano plots for the comparisons performed and differentially expressed features are still highlighted in red. A volcano plot represents the log of the adjusted P value as a function of the log ratio of differential expression. 
 
 
 
 Figure 14: Volcano plot(s) of each comparison. Red dots represent significantly differentially expressed features. 
 
 
 Full results as well as lists of differentially expressed features are provided in the following text files which can be easily read in a spreadsheet. For each comparison: 
 
 TestVsRef.complete.txt contains results for all the features; 
 TestVsRef.up.txt contains results for significantly up-regulated features. Features are ordered from the most significant adjusted p-value to the less significant one; 
 TestVsRef.down.txt contains results for significantly down-regulated features. Features are ordered from the most significant adjusted p-value to the less significant one. 
 
 These files contain the following columns: 
 
 Id: unique feature identifier; 
 sampleName: raw counts per sample; 
 norm.sampleName: rounded normalized counts per sample; 
 baseMean: base mean over all samples; 
 C1, C2, C3, C4, C5, C6 and C7: means (rounded) of normalized counts of the biological conditions; 
 FoldChange: fold change of expression, calculated as  \(2^{\log_2(\text{FC})}\) ; 
 log2FoldChange:  \(\log_2(\text{FC})\)  as estimated by the GLM model. It reflects the differential expression between Test and Ref and can be interpreted as  \(\log_2(\frac{\text{Test}}{\text{Ref}})\) . If this value is:
 
 around 0: the feature expression is similar in both conditions; 
 positive: the feature is up-regulated ( \(\text{Test} &gt; \text{Ref}\) ); 
 negative: the feature is down-regulated ( \(\text{Test} &lt; \text{Ref}\) ); 
  
 pvalue: raw p-value from the statistical test; 
 padj: adjusted p-value on which the cut-off  \(\alpha\)  is applied; 
 dispGeneEst: dispersion parameter estimated from feature counts (i.e. black dots on figure 11); 
 dispFit: dispersion parameter estimated from the model (i.e. red dots on figure 11); 
 dispMAP: dispersion parameter estimated from the Maximum  A Posteriori  model; 
 dispersion: final dispersion parameter used to perform the test (i.e. blue dots and circles on figure 11); 
 betaConv: convergence of the coefficients of the model (TRUE or FALSE); 
 maxCooks: maximum Cook’s distance of the feature. 
 
 
 
 
  6  R session information and parameters 
 The versions of the R software and Bioconductor packages used for this analysis are listed below. It is important to save them if one wants to re-perform the analysis in the same conditions. 
 
 R version 3.4.1 (2017-06-30), x86_64-pc-linux-gnu 
 
 Locale: LC_CTYPE=en_US.UTF-8, LC_NUMERIC=C, LC_TIME=en_US.UTF-8, LC_COLLATE=en_US.UTF-8, LC_MONETARY=en_US.UTF-8, LC_MESSAGES=en_US.UTF-8, LC_PAPER=en_US.UTF-8, LC_NAME=C, LC_ADDRESS=C, LC_TELEPHONE=C, LC_MEASUREMENT=en_US.UTF-8, LC_IDENTIFICATION=C 
 
 Running under: CentOS Linux 7 (Core) 
 
 Matrix products: default 
 
 BLAS: /shared/mfs/data/software/miniconda/envs/eba2018_rnaseq_ref/lib/R/lib/libRblas.so 
 
 LAPACK: /shared/mfs/data/software/miniconda/envs/eba2018_rnaseq_ref/lib/R/lib/libRlapack.so 
 
 Base packages: base, datasets, graphics, grDevices, methods, parallel, stats, stats4, utils 
 
 Other packages: Biobase 2.40.0, BiocGenerics 0.26.0, BiocParallel 1.14.2, DelayedArray 0.6.6, DESeq2 1.18.1, edgeR 3.22.5, GenomeInfoDb 1.16.0, GenomicRanges 1.32.7, IRanges 2.14.12, limma 3.36.5, matrixStats 0.54.0, S4Vectors 0.18.3, SARTools 1.6.3, SummarizedExperiment 1.10.1, xtable 1.8-3 
 
 Loaded via a namespace (and not attached): acepack 1.4.1, annotate 1.58.0, AnnotationDbi 1.42.1, backports 1.1.2, base64enc 0.1-3, bit 1.1-12, bit64 0.9-7, bitops 1.0-6, blob 1.1.1, checkmate 1.8.5, cluster 2.0.7-1, colorspace 1.3-2, compiler 3.4.1, crayon 1.3.4, data.table 1.11.4, DBI 1.0.0, digest 0.6.18, evaluate 0.11, foreign 0.8-71, Formula 1.2-3, genefilter 1.62.0, geneplotter 1.58.0, GenomeInfoDbData 1.1.0, ggplot2 3.0.0, grid 3.4.1, gridExtra 2.3, gtable 0.2.0, Hmisc 4.1-1, htmlTable 1.12, htmltools 0.3.6, htmlwidgets 1.2, knitr 1.20, lattice 0.20-35, latticeExtra 0.6-28, lazyeval 0.2.1, locfit 1.5-9.1, magrittr 1.5, Matrix 1.2-14, memoise 1.1.0, munsell 0.5.0, nnet 7.3-12, pillar 1.3.0, plyr 1.8.4, RColorBrewer 1.1-2, Rcpp 1.0.0, RCurl 1.95-4.11, rlang 0.3.0.1, rmarkdown 1.10, rpart 4.1-13, rprojroot 1.3-2, RSQLite 2.1.1, rstudioapi 0.7, scales 1.0.0, splines 3.4.1, stringi 1.2.4, stringr 1.3.1, survival 2.42-6, tibble 1.4.2, tools 3.4.1, XML 3.98-1.16, XVector 0.20.0, yaml 2.2.0, zlibbioc 1.26.0 
 
 Parameter values used for this analysis are: 
 
 workDir: /shared/projects/strepthost/DESEQanalyses/DEseq24 
 projectName: Streptomics 
 author: Stéphanie Bury-Moné 
 targetFile: targetDESEQ24.txt 
 rawDir: /shared/projects/strepthost/DESEQanalyses/counts_WT_AOUT2020/ 
 featuresToRemove: alignment_not_unique, ambiguous, no_feature, not_aligned, too_low_aQual 
 varInt: Condition 
 condRef: C1 
 batch: NULL 
 fitType: parametric 
 cooksCutoff: TRUE 
 independentFiltering: TRUE 
 alpha: 0.05 
 pAdjustMethod: BH 
 typeTrans: VST 
 locfunc: median 
 colors: #FF0000FF, #FFDB00FF, #49FF00FF, #00FF92FF, #0092FFFF, #4900FFFF, #FF00DBFF 
 
 
 
 Bibliography 
 
 
 1. R Core Team.  R: A language and environment for statistical computing . Vienna, Austria : R Foundation for Statistical Computing, 2017 : 
 
 
 2. Gentleman RC, Carey VJ, Bates DM  et al.  Bioconductor: Open software development for computational biology and bioinformatics.  Genome Biology  2004 ; 5: R80. 
 
 
 3. Anders S, Huber W. Differential expression analysis for sequence count data.  Genome Biology  2010 ; 11: R106. 
 
 
 4. Love MI, Huber W, Anders S. Moderated estimation of fold change and dispersion for rna-seq data with deseq2.  Genome Biology  2014 ; 15: 550. 
 
 
 5. Schulze SK, Kanwar R, Gölzenleuchter M  et al.  SERE: Single-parameter quality control and sample comparison for rna-seq.  BMC Genomics  2012 ; 13: 524. 
 
 
 6. Cook RD. Detection of influential observation in linear regression.  Technometrics  1977 ; 19: 15–18. 
 
 
 7. Cox DR, Reid N. Parameter orthogonality and approximate conditional inference.  Journal of the Royal Statistical Society. Series B (Methodological)  1987 ; 49: 1–39. 
 
 
 8. Benjamini Y, Hochberg Y. Controlling the false discovery rate: A practical and powerful approach to multiple testing.  Journal of the Royal Statistical Society. Series B (Methodological)  1995 ; 57: 289–300. 
 
 
 


 
 

 

 

 
 

 
 
