# Supplementary material for: Dynamics of the compartmentalized Streptomyces chromosome during metabolic differentiation
Source: Nat Commun. 2021 Sep 1;12:5221. doi: 10.1038/s41467-021-25462-1 (PMC8410849; doi:10.1038/s41467-021-25462-1)
Supplement: Supplementary file 8 — Reporting Summary [file 41467_2021_25462_MOESM8_ESM.pdf]

nature portfolio

Corresponding author(s): Stéphanie Bury-Moné, Virginia Lioy

Last updated by author(s): Jul 10, 2021

Reporting Summary

Nature Portfolio wishes to improve the reproducibility of the work that we publish. This form provides structure for consistency and transparency in reporting. For further information on Nature Portfolio policies, see our Editorial Policies and the Editorial Policy Checklist.

Statistics

For all statistical analyses, confirm that the following items are present in the figure legend, table legend, main text, or Methods section.

n/a	Confirmed	
☐	☒	The exact sample size (*n*) for each experimental group/condition, given as a discrete number and unit of measurement
☐	☒	A statement on whether measurements were taken from distinct samples or whether the same sample was measured repeatedly
☐	☒	The statistical test(s) used AND whether they are one- or two-sided<br>*Only common tests should be described solely by name; describe more complex techniques in the Methods section.*
☐	☒	A description of all covariates tested
☐	☒	A description of any assumptions or corrections, such as tests of normality and adjustment for multiple comparisons
☐	☒	A full description of the statistical parameters including central tendency (e.g. means) or other basic estimates (e.g. regression coefficient) AND variation (e.g. standard deviation) or associated estimates of uncertainty (e.g. confidence intervals)
☐	☒	For null hypothesis testing, the test statistic (e.g. *F*, *t*, *r*) with confidence intervals, effect sizes, degrees of freedom and *P* value noted<br>*Give P values as exact values whenever suitable.*
☒	☐	For Bayesian analysis, information on the choice of priors and Markov chain Monte Carlo settings
☐	☒	For hierarchical and complex designs, identification of the appropriate level for tests and full reporting of outcomes
☐	☒	Estimates of effect sizes (e.g. Cohen's *d*, Pearson's *r*), indicating how they were calculated

Our web collection on statistics for biologists contains articles on many of the points above.

Software and code

Policy information about availability of computer code

Data collection

rast Classic pipeline (FIGfam version: release 70)
BLASTP 2.11.0+
python 3.8.5
R 4.0.3
Rstudio 1.3.01093
http://bim.i2bc.paris-saclay.fr/synteruptor/

Data analysis

rast Classic pipeline (FIGfam version: release 70)
BLASTP 2.11.0+
python 3.8.5
R 4.0.3
Rstudio 1.3.01093
Matlab (2015, 2020, 2021)
antiSMASH5.1.0
STAR software (v2.5.4)
featureCounts program (v2.0.1)
SARTools (Statistical Analysis of RNA-Seq data Tools, v1.6.3)
FactoShiny R package (v2.4)
Bowtie 2 (v2.2.3)
The scripts used for data analyses are available on the following Github links: RNA-seq analyses (https://github.com/PF2-pasteur-fr/SARTools), 3C-seq contact-map analyses (https://github.com/koszullab/E_coli_analysis), 3C-seq frontier index analyses (https://github.com/VickyTche/Frontier_Index_Streptomyces.git; https://osf.io/a23de/; DOI 10.17605/OSF.IO/A23DE), persistence and core genome analyses (https://github.com/jnlorenzi/pipeline-core-and-persistence; DOI : 10.5281/zenodo.5067212), Synteruptor software associated code (https://

github.com/jnlorenzi/synteruptor; DOI: 10.5281/zenodo.5080081).

For manuscripts utilizing custom algorithms or software that are central to the research but not yet described in published literature, software must be made available to editors and reviewers. We strongly encourage code deposition in a community repository (e.g. GitHub). See the Nature Portfolio guidelines for submitting code & software for further information.

Data

Policy information about availability of data

All manuscripts must include a data availability statement. This statement should provide the following information, where applicable:

- Accession codes, unique identifiers, or web links for publicly available datasets
- A description of any restrictions on data availability
- For clinical datasets or third party data, please ensure that the statement adheres to our policy

The RNA-seq and 3C-seq data generated during in this study have been deposited in the NCBI Gene Expression Omnibus (GEO, https://www.ncbi.nlm.nih.gov/geo/) under the accession code GSE162865. (https://www-ncbi-nlm-nih-gov.insb.bib.cnrs.fr/geo/query/acc.cgi?acc=GSE162865). The list of the core genome CDSs and the persistence index values are available in Supplementary Data 3. Source data are provided with this paper (to generate Supplementary Fig.2b & c).

Field-specific reporting

Please select the one below that is the best fit for your research. If you are not sure, read the appropriate sections before making your selection.

☒ Life sciences ☐ Behavioural & social sciences ☐ Ecological, evolutionary & environmental sciences

For a reference copy of the document with all sections, see nature.com/documents/nr-reporting-summary-flat.pdf

Life sciences study design

All studies must disclose on these points even when the disclosure is negative.

Sample size	For the RNA-seq analyses, three independent experiments (performed on different days) were carried out for each studied condition, except for C3, C9 and C10 that were performed in duplicate and C5 in quadruple. The statistical significance of RNA-seq analysis was assessed using the SARTools DESeq2-based pipeline (see Statistical Report provided in Supplementary Data 4). We ensured that the replicates clustered according to the biological conditions (and not to an experimental biais). For the 3C-seq analyses, two independent experiments (performed on different days) were carried out for each condition. The number of replicates is rather classical for this type of experiments, for which the cost influences the sample size.
Data exclusions	No data exclusion
Replication	All experiments were replicated. We used data exploration (Supplementary Data 4) to control the quality of the replicates.
Randomization	Sample collection and measurements were performed independently (on different days, with different medium batchs), in order to minimize experimental biais. Our experimentations did not involve allocation of samples into experimental groups, since we conducted experiments based on the harvesting of bacterial cultures during growth in different media.
Blinding	To monitor chromosome conformation, transcriptiome and antibiotic production over growth, complete blinding is impossible. Therefore, investigators were diligent about adhering to the same procedures and keeping the same standard for sample collection and analysis. Sequencing was conducted in a blinded fashion (i.e. people in charge of sequencing did not have any biological information concerning the samples).

Reporting for specific materials, systems and methods

We require information from authors about some types of materials, experimental systems and methods used in many studies. Here, indicate whether each material, system or method listed is relevant to your study. If you are not sure if a list item applies to your research, read the appropriate section before selecting a response.

Materials & experimental systems

n/a	Involved in the study	
☒	☐	Antibodies
☒	☐	Eukaryotic cell lines
☒	☐	Palaeontology and archaeology
☒	☐	Animals and other organisms
☒	☐	Human research participants
☒	☐	Clinical data
☒	☐	Dual use research of concern

Methods

n/a	Involved in the study	
☒	☐	ChIP-seq
☒	☐	Flow cytometry
☒	☐	MRI-based neuroimaging
